# Frame-Theoretic AOA Network Augmentation with the Minimum Number of Additional Sensors and Optimal Bearings

**DOI:** 10.3390/s26175626

**Published:** 2026-09-04

**Authors:** Kun Liu, Xinpeng Fang, Junfang Li, Yangmei Zhang

**Affiliations:** 1 School of Electrical and Information Engineering, Xihang University, Xi’an 710077, China; li_jf@xaau.edu.cn (J.L.); 201707019@xaau.edu.cn (Y.Z.); 2 School of Aerospace Science and Technology, Xidian University, Xi’an 710071, China; xinpengfang@163.com

**Keywords:** angle-of-arrival localization, sensor augmentation, sensor geometry, Fisher information matrix, Cramér–Rao lower bound, frame theory

## Abstract

In line-of-sight sensing and surveillance systems, angle-of-arrival (AOA) localization accuracy depends strongly on sensor geometry. The spatially distributed sensors considered here may be electromagnetic, acoustic, optical, or other directional sensing devices that provide bearing measurements with known nominal accuracy. Existing frame-theoretic sensor-augmentation studies usually optimize the added-sensor bearings for a fixed number of sensors, whereas practical network design often requires determining the minimum number of sensors needed to satisfy a prescribed accuracy constraint. Poorly balanced bearing geometry can make the Fisher information matrix (FIM) ill-conditioned and increase localization uncertainty. This paper addresses this problem using the A-optimal Cramér–Rao lower bound trace as the accuracy measure, because it represents the sum of the lower bounds on the coordinate estimation-error variances. The existing Fisher information matrix is described by its total information, strong information direction, and eigenvalue gap. Each additional sensor is represented as a weighted vector in a double-angle plane, which converts the bearing-design problem into a planar vector-balancing problem. This representation separates the effects of total information and directional imbalance on localization performance. A polygon condition is used to determine the minimum achievable imbalance and A-optimal cost for fixed sensor weights. For equal-weight sensors, a direct rule is derived for finding the minimum required number of additional sensors, together with explicit bearing constructions for weak-direction compensation and tight-frame completion. The analysis is also extended to unequal sensor weights. Numerical optimization and Monte Carlo localization experiments confirm the analytical results and show that the proposed method improves information balance, FIM conditioning, empirical localization accuracy, and tail-error performance compared with the reference configurations. Random-network and target-position mismatch experiments further evaluate the design beyond the nominal network geometry.

## 1. Introduction

Accurate location information has become an essential component of wireless sensing, autonomous systems, the industrial Internet of Things, and integrated communication-sensing networks. Among the available measurement modalities, the angle of arrival (AOA) is particularly attractive because it supports passive or asynchronous localization without requiring precise time synchronization between a target and spatially distributed sensors. AOA measurements have therefore been used in radar surveillance, wireless positioning, cooperative sensing, indoor localization, and underwater monitoring [1,2,3].

Recent studies have improved AOA acquisition and localization in multipath environments, indoor scenarios, moving-target applications, and three-dimensional localization problems [4,5,6,7,8]. These developments primarily address measurement processing and estimator design. However, because each AOA measurement provides inherently directional information, localization accuracy remains strongly affected by the spatial distribution of the sensors. Here, sensor geometry denotes the relative spatial configuration between the target and the sensors, especially the bearings, target–sensor distances, and angular distribution of the sensing directions. Measurement quality, the number of sensors, and sensor–target geometry must therefore be considered jointly when designing an AOA localization network.

The Fisher information matrix (FIM) and the Cramér–Rao lower bound (CRLB) provide an estimator-independent framework for quantifying this geometric effect. In AOA localization, each sensor contributes a rank-one information matrix whose magnitude depends on its angular accuracy and target distance and whose information direction is perpendicular to the corresponding line of sight (LOS). Sensors with similar bearings consequently provide highly redundant information, whereas complementary information directions improve local observability and reduce error anisotropy. Previous studies have characterized optimal angular separation, sensor–target geometry, the eigenvalue distribution of the FIM, and AOA localization accuracy in two- and three-dimensional settings [9,10,11,12,13]. These results demonstrate the importance of incorporating sensor geometry explicitly into information-based localization design.

Information-based sensor placement is commonly formulated by optimizing a scalar function of the FIM or its inverse. The FIM summarizes the local information provided by sensor geometry and measurement accuracy, while its inverse provides the CRLB matrix that lower-bounds the covariance of any unbiased localization estimator. The A-optimal criterion minimizes the trace of the inverse FIM, which equals the sum of the CRLBs on the coordinate-wise error variances. The D-optimal criterion maximizes the determinant of the FIM, which is equivalent to reducing the area of the CRLB-based uncertainty ellipse, whereas the E-optimal criterion maximizes its smallest eigenvalue and improves the worst-direction information. Because A-optimality has a direct interpretation in terms of the total position-error variance lower bound, it is adopted in this study. These criteria indicate that accumulating sufficient information and distributing it effectively across spatial directions are related but distinct design objectives. Information-based geometry design has been investigated for both dynamic angle-only trajectory optimization and static three-dimensional AOA sensor placement [14,15].

The above information-based criteria explain how a given sensor geometry affects localization accuracy, but practical deployment must also account for sensing quality and feasible deployment locations. The information supplied by a sensor depends on its target distance, measurement modality, and hardware accuracy, while deployment may be restricted by boundaries, obstacles, communication requirements, or installation conditions. Existing studies have addressed placement using fused TOA-RSS-AOA measurements [16], source-position uncertainty [17], known signal structures [18], unified numerical optimization [19], and deployment-region constraints [20]. Although these methods provide substantial modeling flexibility, the resulting formulations are generally nonconvex and may require iterative computation, careful initialization, or repeated solution under different deployment settings.

Besides continuous placement, determining how many sensors should be deployed or selected is also important for controlling network cost under a prescribed performance requirement. Joint sensor selection and deployment based on optimal sensor–target geometry was investigated in [21], while multi-target placement with coupled sensor clusters was formulated in [22]. Sensor-position and velocity design for TDOA-FDOA tracking under sensor errors and distance-dependent noise was studied in [23]. Related geometry-design principles have been extended to receiver placement for multistatic localization [24], simultaneous sensor-and-source localization [25], and full-set TDOA systems with sensor-location errors [26]. For mobile and networked platforms, cooperative UAV placement for multistatic TOA localization was optimized in [27]. These studies extend sensor-deployment design to sensor selection, multiple targets, platform mobility, and system uncertainty. Nevertheless, they largely address complete network design, selection from a finite set of available sensors, or geometry optimization for a predetermined number of sensors.

Frame theory analyzes redundant vector systems through their frame operators, which are formed by summing the outer products of the frame vectors [28]. In AOA localization, the weighted sensor information directions can be treated as frame vectors, and their frame operator coincides with the FIM, thereby linking information balance directly to sensor geometry. For unit-norm frames with a prescribed number of vectors, the relationship between frame-potential minimization and finite normalized tight frames was established in [29]. Prescribed-norm frame completion has also been studied in terms of convex-potential optimality, local and global minimizers, and computational construction [30,31]. However, these studies do not directly address the threshold-based problem of determining the number of additional sensors in AOA localization networks. When the weighted information vectors form a tight frame, the FIM is proportional to the identity matrix, and the information is equally distributed between orthogonal directions. Sensor-geometry design can therefore be interpreted as a directional vector-balancing problem. This correspondence was used to characterize optimal localization geometries in [32]. Optimal placement for networks containing both fixed and controllable sensors was studied in [33], and AOA sensor augmentation was formulated as a frame-completion problem in [34], where optimal bearings were derived for a prescribed number of additional sensors. The polygon-closure condition and fixed-size minimum-residual result used here are explicit two-dimensional AOA formulations of established frame-completion principles. The contribution of this work is to combine these formulations with a prescribed A-optimal threshold to determine the minimum number of additional sensors that satisfy the accuracy requirement and their corresponding bearings.

Existing frame-theoretic augmentation results assume that the number of additional sensors is known in advance. For an installed network that fails to satisfy a new accuracy requirement, however, the designer must determine both the minimum number of additional sensors and their bearing configuration. Too few sensors cannot satisfy the requirement, whereas unnecessary sensors increase deployment and maintenance costs. Tight-frame completion alone cannot determine this number because it guarantees optimal directional balance for a given amount of total information but does not ensure satisfaction of the prescribed CRLB-trace threshold. Moreover, before tight completion becomes feasible, compensating for the weak information direction of the existing network can be optimized. The augmentation design must therefore account jointly for information accumulation, directional imbalance, the number of sensors, and bearing geometry.

Accordingly, this paper studies the augmentation of a two-dimensional AOA localization network with fixed existing sensors under an A-optimal CRLB-trace constraint. The existing FIM is characterized by its total information, principal eigendirection, and eigenvalue gap. Each added sensor is represented by a weighted vector in the double-angle plane, converting bearing optimization into a planar vector-balancing problem. The generalized polygon inequality then gives the minimum residual imbalance for a given number of sensors, enabling direct determination of the minimum required number and, for equal-weight sensors, explicit optimal bearing construction. This analysis avoids repeated nonlinear optimization and links the accuracy threshold, sensor weights, required number of sensors, and optimal geometry.

The main contributions of this study are summarized as follows:

- An exact frame-theoretic decomposition of the augmented A-optimal cost is derived, separating the effect of total accumulated information from the penalty caused by residual directional imbalance.
- For prescribed additional-sensor information weights, the generalized polygon inequality is specialized to the two-dimensional AOA double-angle representation to obtain the minimum attainable residual imbalance and A-optimal cost. The result applies to both equal- and unequal-weight sensors and allows the minimum achievable cost to be evaluated without nonlinear bearing optimization for a prescribed number of additional sensors.
- For equal-weight sensors, a closed-form rule is developed to determine the minimum number of additional sensors required to satisfy a prescribed CRLB-trace threshold. Explicit optimal bearing constructions are provided for the weak-direction compensation and tight-frame completion stages.
- The analytical results are validated through multistart numerical optimization, Monte Carlo AOA localization, unequal-weight experiments, random-network tests, and target-position mismatch studies.

The remainder of this paper is organized as follows. Section 2 presents the AOA measurement model, establishes the frame representation of the existing sensor network, and formulates the accuracy-constrained augmentation problem. Section 3 develops the frame-theoretic performance analysis. Section 4 derives the minimum-sensor augmentation rule and the corresponding optimal bearing constructions. Section 5 verifies the analytical results through numerical optimization and Monte Carlo localization experiments. Finally, the concluding section summarizes the main findings and discusses possible extensions.

## 2. AOA Localization Model and Sensor Augmentation Problem

This section presents the two-dimensional AOA measurement model and describes the information provided by the existing sensor network. The FIM is then connected to finite-dimensional frame theory. Finally, the sensor augmentation problem is formulated to determine the minimum number and optimal bearings of the additional sensors under a prescribed accuracy constraint.

### 2.1. AOA Measurement Model

Consider a two-dimensional AOA localization system consisting of *m* fixed sensors, where each sensor provides a bearing-only measurement of the target relative to its own position. Accordingly, each AOA measurement contributes directional Fisher information that lies perpendicular to the corresponding target–sensor line of sight. Let the nominal target position be $p={\left[x,y\right]}^{T}$, which denotes the reference target location at which the local FIM and CRLB are evaluated, and let the position of the *i*-th sensor be $s_{i}={\left[x_{i},y_{i}\right]}^{T}$, where i=1,2,…,m.

Taking the target position as the reference point, let $\varphi_{i}$ denote the bearing from the target to the *i*-th sensor. Here, the bearing is the direction angle of the target–sensor line measured counterclockwise from the positive *x*-axis. The sensor–target displacement vector can then be written as

(1) $$s_{i}-p=r_{i}\left[\begin{array}{c} \cos\varphi_{i}\\ \sin\varphi_{i} \end{array}\right].$$

The AOA measurement obtained by the *i*-th sensor is modeled as

(2) $${\tilde{\theta }}_{i}=h_{i}\left(p\right)+v_{i},$$

where ${\tilde{\theta }}_{i}$ is the measured AOA, $h_{i}\left(p\right)=\mathrm{atan2}\left(y-y_{i},x-x_{i}\right)$ is the noise-free bearing from the sensor to the target, and $v_{i}$ is the measurement error.

The measurement errors are assumed to be mutually independent and Gaussian, with $v_{i}∼N\left(0,\sigma_{i}^{2}\right)$, where $\sigma_{i}^{2}$ is the AOA measurement-error variance of the *i*-th sensor. Thus, $E[v_{i}v_{k}]=0$ for i≠k. Angular errors and their standard deviations are expressed in radians when computing the FIM. The Gaussian model is used as a local approximation for sufficiently small angular errors, while angular residuals are mapped to a principal interval in the numerical localization procedure. The following FIM analysis is local and is evaluated at the nominal target position p. Accordingly, the derived optimality results apply to the nominal independent-noise FIM. Extensions to correlated noise, systematic bearing bias, and non-Gaussian AOA errors require a performance model that accounts for noise-model mismatch and are left for future work.

Differentiating $h_{i}\left(p\right)$ with respect to the target position gives

(3) $$g_{i}=\nabla_{p}h_{i}\left(p\right)=\frac{1}{r_{i}}\left[\begin{array}{c} \sin\varphi_{i}\\ -\cos\varphi_{i} \end{array}\right].$$

Define the unit information-direction vector as

(4) $$a_{i}=\left[\begin{array}{c} \sin\varphi_{i}\\ -\cos\varphi_{i} \end{array}\right],\;{∥a_{i}∥}_{2}=1.$$

**Figure 1:**
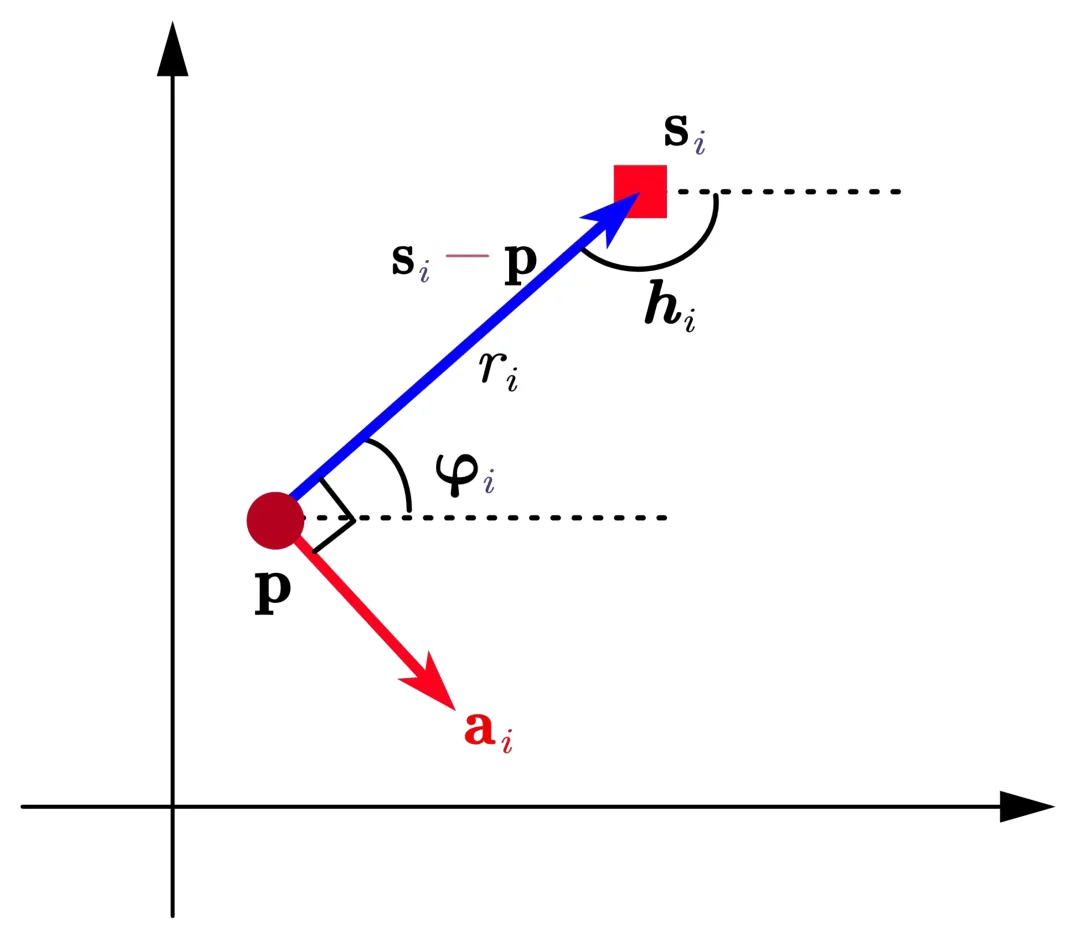
AOA localization geometry for the *i*-th sensor.

The vector $a_{i}$ is perpendicular to the line of sight between the target and the sensor. Therefore, an individual AOA measurement provides local position information only in the direction perpendicular to its line of sight. The AOA geometry and corresponding information-direction vector are illustrated in Figure 1.

As shown in Figure 1, $\phi_{i}$ is the target-to-sensor bearing, $h_{i}\left(p\right)$ is the reverse sensor-to-target bearing, and $a_{i}⊥\left(s_{i}-p\right)$. The reverse bearing only changes the sign of the information-direction vector and therefore leaves $a_{i}a_{i}^{T}$ unchanged.

Under the Gaussian measurement model, the contribution of the *i*-th sensor to the target-position FIM is

(5) $$J_{i}=\frac{1}{\sigma_{i}^{2}}g_{i}g_{i}^{T}=w_{i}a_{i}a_{i}^{T}.$$

Here, $w_{i}=1/\left(\sigma_{i}^{2}r_{i}^{2}\right)$ is the information weight of the *i*-th sensor. A smaller measurement variance or a shorter target–sensor distance gives a larger information weight.

Because $a_{i}a_{i}^{T}$ has rank one, a single AOA sensor cannot determine a two-dimensional target position by itself. Multiple sensors are therefore required to provide information in different directions. If their information directions are highly concentrated, the resulting FIM may be poorly conditioned and the localization error may be strongly direction-dependent.

### 2.2. Frame Representation of the Existing Sensor Network

The frame representation rewrites the directional AOA information as a collection of vectors $\sqrt{w_{i}}a_{i}$. The outer-product sum of these vectors has the same matrix form as the FIM, so frame balance corresponds directly to information balance in localization. Because the measurement errors are mutually independent, the total FIM provided by the existing sensors is

(6) $$J_{0}=\sum_{i=1}^{m}J_{i}=\sum_{i=1}^{m}w_{i}a_{i}a_{i}^{T}.$$

Define the weighted information vector $f_{i}=\sqrt{w_{i}}a_{i}$ and the vector collection $\Phi_{0}=\left\{f_{1},f_{2},\ldots ,f_{m}\right\}$. A finite collection $\Phi ={\left\{f_{i}\right\}}_{i=1}^{m}\subset R^{2}$ is a frame for $R^{2}$ if there exist constants $0<\alpha_{\Phi }\leq \beta_{\Phi }<\infty$ such that [35]

(7) $$\alpha_{\Phi }{∥q∥}_{2}^{2}\leq \sum_{i=1}^{m}{\left|q^{T}f_{i}\right|}^{2}\leq \beta_{\Phi }{∥q∥}_{2}^{2},\;\forall \;q\in R^{2}.$$

The constants $\alpha_{\Phi }$ and $\beta_{\Phi }$ are the lower and upper frame bounds, respectively. In the finite-dimensional case considered here, the collection forms a frame if and only if its vectors span $R^{2}$.

The frame operator associated with $\Phi_{0}$, denoted by $S_{\Phi_{0}}:R^{2}\to R^{2}$, is defined by

(8) $$S_{\Phi_{0}}q=\sum_{i=1}^{m}\left(q^{T}f_{i}\right)f_{i}=\left(\sum_{i=1}^{m}f_{i}f_{i}^{T}\right)q=J_{0}q,\;q\in R^{2}.$$

Therefore, the matrix representation of $S_{\Phi_{0}}$ in the standard basis of $R^{2}$ is exactly the existing FIM $J_{0}$. This connection allows the information distribution of the sensor network to be studied through the directions and lengths of its weighted frame vectors.

The existing network is assumed to be locally observable at the nominal target position. Thus, the vectors in $\Phi_{0}$ span $R^{2}$, and $J_{0}$ is positive definite. Its eigendecomposition is

(9) $$J_{0}=U_{\vartheta }\left[\begin{array}{cc} \kappa_{1} & 0\\ 0 & \kappa_{2} \end{array}\right]U_{\vartheta }^{T}.$$

The eigenvalues are ordered as $\kappa_{1}\geq \kappa_{2}>0$, and

(10) $$U_{\vartheta }=\left[\begin{array}{cc} \cos\vartheta & -\sin\vartheta \\ \sin\vartheta & \cos\vartheta \end{array}\right]$$

is the orthogonal matrix whose first and second columns give the strong and weak information directions, respectively. Accordingly, $\kappa_{1}$ and $\kappa_{2}$ measure the Fisher information along these directions, while ϑ gives the angle of the strong information direction relative to the global *x*-axis.

**Figure 2:**
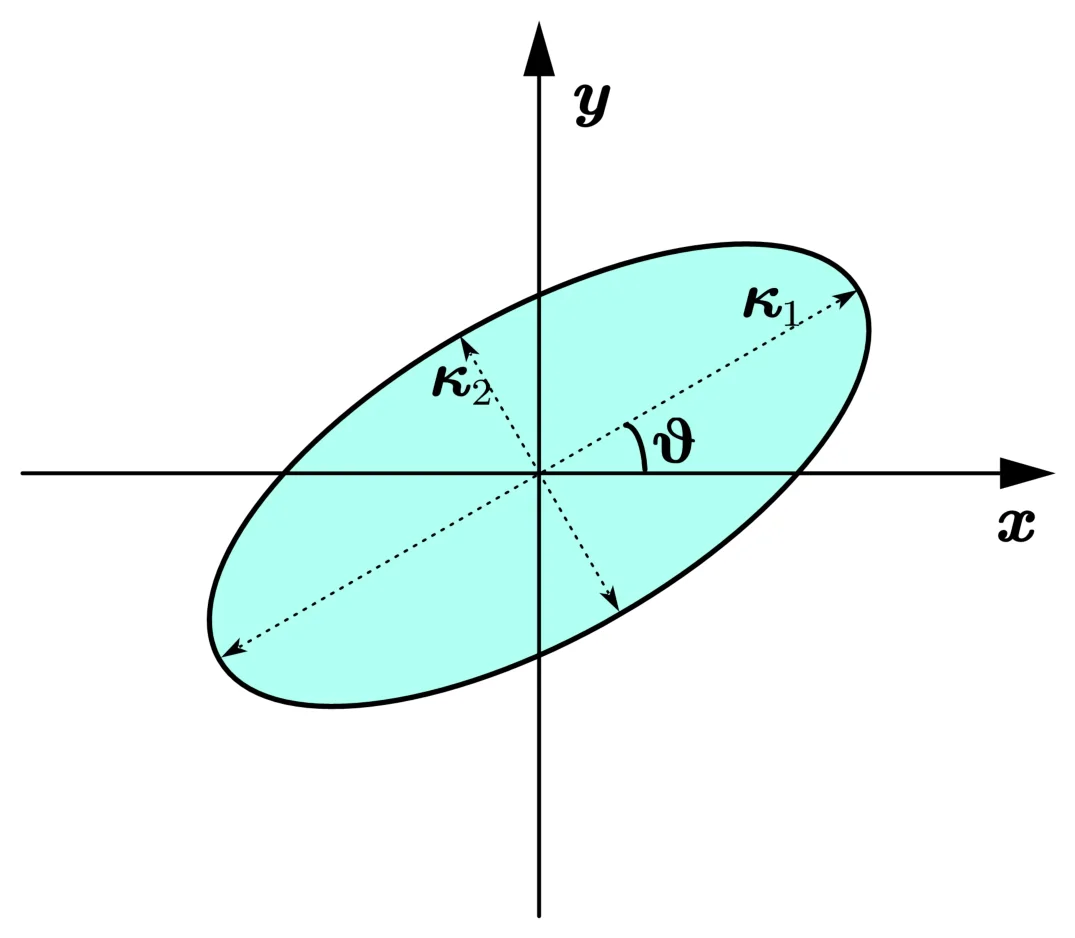
Geometric interpretation of the existing AOA FIM.

The geometric interpretation of this eigenstructure is shown in Figure 2. The major and minor axes of the information ellipse correspond to the strong and weak information directions, respectively.

The exact lower and upper frame bounds of $\Phi_{0}$ are the smallest and largest eigenvalues of its frame operator. Therefore, $\alpha_{\Phi_{0}}=\kappa_{2}$ and $\beta_{\Phi_{0}}=\kappa_{1}$. Their difference measures the imbalance between the two information directions. The FIM condition number is $\mathrm{cond}\left(J_{0}\right)=\kappa_{1}/\kappa_{2}$. A value of one indicates perfectly balanced directional information, whereas a large value indicates strong information anisotropy and poor conditioning.

Define the total information as $S_{0}=\mathrm{tr}\left(J_{0}\right)=\kappa_{1}+\kappa_{2}$ and the eigenvalue gap as $\kappa =\kappa_{1}-\kappa_{2}$. The parameter $S_{0}$ measures the total information provided by the existing network, whereas κ measures the difference between its strong and weak information directions. Since $\kappa_{1}\geq \kappa_{2}>0$, it follows that $0\leq \kappa <S_{0}$.

A frame is tight if its lower and upper frame bounds are equal. Therefore, $\Phi_{0}$ is a tight frame if and only if $\kappa_{1}=\kappa_{2}$, or equivalently, κ=0. Since $\mathrm{tr}\left(J_{0}\right)=S_{0}$, the two eigenvalues are then both equal to $S_{0}/2$, giving $J_{0}=\left(S_{0}/2\right)I_{2}$. In this case, the FIM provides the same amount of information in every direction. When κ>0, the network provides more information along the strong direction than along the weak direction. The corresponding CRLB error ellipse is therefore longer along the weak information direction.

According to the Cramér–Rao inequality, the covariance matrix of any unbiased estimator $\hat{p}$ satisfies $\mathrm{Cov}\left(\hat{p}\right)⪰J_{0}^{-1}$. This study uses the trace of the inverse FIM as the localization performance criterion:

(11) $$A_{0}=\mathrm{tr}\left(J_{0}^{-1}\right)=\frac{1}{\kappa_{1}}+\frac{1}{\kappa_{2}}=\frac{4S_{0}}{S_{0}^{2}-\kappa^{2}}.$$

The quantity $A_{0}$ has units of squared distance and represents the sum of the coordinate-wise position-error variance lower bounds. The corresponding equivalent CRLB RMSE is defined as ${\mathrm{RMSE}}_{\mathrm{CRLB},0}=\sqrt{A_{0}}$.

For a fixed total information $S_{0}$, (11) gives $A_{0}\geq 4/S_{0}$, where equality holds if and only if κ=0. Thus, among all two-dimensional FIMs with the same trace, a tight-frame FIM minimizes the A-optimal CRLB trace. This result also shows that localization performance depends on both the total information and its directional balance.

### 2.3. Accuracy-Constrained Sensor Augmentation Model

The augmentation problem therefore centers around adding weighted AOA information vectors with properly chosen bearings so that the completed FIM satisfies the prescribed A-optimal CRLB-trace constraint. Suppose that *n* additional AOA sensors are deployed while the existing sensors remain fixed. Let $\phi_{j}$ denote the bearing from the nominal target position to the *j*-th additional sensor. Its unit information-direction vector is

(12) $$b_{j}=\left[\begin{array}{c} \sin\phi_{j}\\ -\cos\phi_{j} \end{array}\right],\;{∥b_{j}∥}_{2}=1,\;j=1,2,\ldots ,n.$$

The main model considers additional sensors with the same AOA measurement-error standard deviation $\sigma_{a}$ and the same target–sensor distance $r_{a}$. Each additional sensor then has information weight $w=1/(\sigma_{a}^{2}r_{a}^{2})$. For a prescribed $r_{a}$ and a physical bearing $\phi_{j}\in \left[0,2\pi \right)$, the position of the *j*-th additional sensor is

(13) $$s_{j}^{\left(a\right)}=p+r_{a}\left[\begin{array}{c} \cos\phi_{j}\\ \sin\phi_{j} \end{array}\right].$$

The bearings $\phi_{j}$ and $\phi_{j}+\pi$ produce the same outer product $b_{j}b_{j}^{T}$, although they correspond to two physical sensor positions on opposite sides of the target. Therefore, the bearing optimization may be restricted to [0,π), while either physical position may be selected during deployment.

After adding *n* equal-weight sensors, the augmented FIM is

(14) $$J_{n}=J_{0}+w\sum_{j=1}^{n}b_{j}b_{j}^{T}.$$

Because $J_{0}$ is positive definite and each additional contribution is positive semidefinite, $J_{n}$ is positive definite for every n≥0.

The A-optimal cost after augmentation is defined as $A_{n}=\mathrm{tr}\left(J_{n}^{-1}\right)$. Let ε>0 be the prescribed upper bound on the A-optimal CRLB trace. The corresponding accuracy constraint is $A_{n}\leq \varepsilon$.

The threshold ε has units of squared distance, and $\sqrt{\varepsilon }$ is the corresponding equivalent CRLB RMSE threshold. Since the CRLB is an estimator-independent lower bound, this constraint is an information-based design condition rather than a guarantee of the finite-sample error of a particular estimator.

For a fixed number *n* of additional sensors, different bearing configurations generally produce different augmented FIMs and A-optimal costs. The minimum achievable cost is defined as

(15) $$A_{\min}\left(n\right)=\min_{\begin{array}{c} 0\leq \phi_{j}<\pi ,\\ j=1,2,\ldots ,n \end{array}}\;\mathrm{tr}\left[{\left(J_{0}+w\sum_{j=1}^{n}b_{j}b_{j}^{T}\right)}^{-1}\right].$$

For n=0, define $A_{\min}\left(0\right)=A_{0}$. The minimum number of additional sensors required to satisfy the prescribed accuracy constraint is

(16) $$n^{★}=\min\left\{n\in N_{0}:A_{\min}\left(n\right)\leq \varepsilon \right\},$$

where $N_{0}=\left\{0,1,2,\ldots \right\}$. If $A_{0}\leq \varepsilon$, then $n^{★}=0$, and the existing network requires no augmentation. Otherwise, $n^{★}\geq 1$. For $n^{★}\geq 1$, any bearing vector attaining $A_{\min}\left(n^{★}\right)$ is an optimal configuration and is denoted by $\phi^{★}={\left[\phi_{1}^{★},\phi_{2}^{★},\ldots ,\phi_{n^{★}}^{★}\right]}^{T}$. The optimal configuration need not be unique.

The required number of additional sensors depends on both the amount and the directional distribution of the added information. Too few sensors may provide insufficient total information, whereas poorly distributed information may lead to unsatisfactory performance even when its total amount is sufficient. The number of additional sensors and bearing geometry must therefore be designed together.

## 3. Frame-Theoretic Analysis of Sensor Augmentation Performance

This section converts the matrix-based bearing-optimization problem into a planar vector-balancing problem by using the eigenvector coordinate system of the existing FIM and the double-angle representation. The eigenvalue imbalance of the existing FIM and the additional AOA sensor contributions are then represented as weighted vectors. This representation separates the effects of total information and directional imbalance and leads to a closed-form expression for the best performance achievable with a fixed number of additional sensors.

### 3.1. Weighted Frame Representation of the Augmented Geometry

To cover sensors with different measurement accuracies or target distances, let $w_{j}>0$ denote the information weight of the *j*-th additional sensor. If its AOA measurement-error standard deviation and target distance are $\sigma_{j}$ and $r_{j}$, respectively, then $w_{j}=1/\left(\sigma_{j}^{2}r_{j}^{2}\right)$. The augmented FIM is

(17) $$J_{n}=J_{0}+\sum_{j=1}^{n}w_{j}b_{j}b_{j}^{T}.$$

The equal-weight model in Section 2.3 is obtained by setting $w_{j}=w$ for all *j*.

Let ϑ denote the strong information direction of the existing FIM, and define the relative bearing of the *j*-th additional sensor as ${\bar{\phi }}_{j}=\phi_{j}-\vartheta$. Transforming its unit information-direction vector into the eigenvector coordinate system of $J_{0}$ gives

(18) $${\bar{b}}_{j}=U_{\vartheta }^{T}b_{j}=\left[\begin{array}{c} \sin{\bar{\phi }}_{j}\\ -\cos{\bar{\phi }}_{j} \end{array}\right].$$

When ${\bar{\phi }}_{j}\equiv 0\;\left(\mathrm{mod}\;\pi \right)$, the bearing from the target to the additional sensor is aligned with the strong information direction of the existing network. Since the AOA information direction is perpendicular to the corresponding LOS, the sensor then adds information to the weak direction. When ${\bar{\phi }}_{j}\equiv \pi /2\;\left(\mathrm{mod}\;\pi \right)$, it adds information to the strong direction.

In the eigenvector coordinate system, the augmented FIM becomes

(19) $${\bar{J}}_{n}=U_{\vartheta }^{T}J_{n}U_{\vartheta }=\left[\begin{array}{cc} a_{n} & c_{n}\\ c_{n} & d_{n} \end{array}\right],$$

where $a_{n}=\kappa_{1}+\sum_{j=1}^{n}w_{j}\sin^{2}{\bar{\phi }}_{j}$, $d_{n}=\kappa_{2}+\sum_{j=1}^{n}w_{j}\cos^{2}{\bar{\phi }}_{j}$, and $c_{n}=-\sum_{j=1}^{n}w_{j}\sin{\bar{\phi }}_{j}\cos{\bar{\phi }}_{j}$.

An orthogonal coordinate transformation does not change the trace, determinant, or eigenvalues of a matrix. Therefore, $J_{n}$ and ${\bar{J}}_{n}$ have the same localization performance. Their common trace is

(20) $$S_{n}=\mathrm{tr}\left(J_{n}\right)=\mathrm{tr}\left({\bar{J}}_{n}\right)=S_{0}+\sum_{j=1}^{n}w_{j}.$$

For a fixed number of sensors and fixed information weights, $S_{n}$ does not depend on the sensor bearings. Changing the bearings only changes how this information is distributed between directions.

Using the double-angle identities, the difference between the two directional information components can be written as

(21) $$\left[\begin{array}{c} a_{n}-d_{n}\\ 2c_{n} \end{array}\right]=\left[\begin{array}{c} \kappa \\ 0 \end{array}\right]-\sum_{j=1}^{n}w_{j}\left[\begin{array}{c} \cos(2{\bar{\phi }}_{j})\\ \sin(2{\bar{\phi }}_{j}) \end{array}\right].$$

Equation (21) maps the relative bearing ${\bar{\phi }}_{j}$, which is defined modulo π, to the direction $2{\bar{\phi }}_{j}$, which is defined modulo 2π. The resulting geometric representation is called the double-angle plane. It is a mathematical tool for bearing design and does not represent the physical sensor positions.

In the double-angle plane, the original eigenvalue gap κ is represented by a fixed vector of length κ along the positive horizontal axis. The *j*-th additional sensor is represented by a vector of length $w_{j}$ and direction $2{\bar{\phi }}_{j}$. For shorter notation, define

(22) $$z_{n}=\kappa -\sum_{j=1}^{n}w_{j}\exp\left(i\;2{\bar{\phi }}_{j}\right),\;i=\sqrt{-1}.$$

The magnitude of $z_{n}$ is $\rho_{n}=\left|z_{n}\right|=\sqrt{{\left(a_{n}-d_{n}\right)}^{2}+4c_{n}^{2}}$. Thus, bearing design is equivalent to choosing the directions of the additional vectors so that their vector sum is as close as possible to the original imbalance vector. The remaining difference has magnitude $\rho_{n}$.

### 3.2. Decomposition of the A-Optimal Cost

The two eigenvalues of the augmented FIM are

(23) $$\lambda_{n,1}=\frac{S_{n}+\rho_{n}}{2},\;\lambda_{n,2}=\frac{S_{n}-\rho_{n}}{2}.$$

Therefore, $\rho_{n}=\lambda_{n,1}-\lambda_{n,2}$ is the eigenvalue gap of the augmented FIM. Since $J_{n}$ is positive definite, its eigenvalues are positive and $0\leq \rho_{n}<S_{n}$.

The A-optimal cost can be expressed as

(24) $$A_{n}=\mathrm{tr}\left(J_{n}^{-1}\right)=\frac{1}{\lambda_{n,1}}+\frac{1}{\lambda_{n,2}}=\frac{4S_{n}}{S_{n}^{2}-\rho_{n}^{2}}.$$

Define the normalized information imbalance as $\xi_{n}=\rho_{n}/S_{n}$, where $0\leq \xi_{n}<1$. Substituting this definition into (24) gives

(25) $$A_{n}=\frac{4}{S_{n}}\frac{1}{1-\xi_{n}^{2}}.$$

Equation (25) separates the effects of total information and directional imbalance. The factor $4/S_{n}$ is the smallest CRLB trace attainable for the given total information, whereas $1/(1-\xi_{n}^{2})\geq 1$ is the increase in cost caused by directional imbalance. When $\xi_{n}=0$, the two eigenvalues are equal and the minimum cost $4/S_{n}$ is attained. As $\xi_{n}$ increases, the eigenvalue gap becomes larger and the A-optimal cost increases. When $\xi_{n}$ approaches one, the smaller eigenvalue approaches zero and the localization uncertainty along the weak information direction becomes large.

Thus, sensor augmentation improves localization performance by increasing the total information $S_{n}$ and reducing the normalized imbalance $\xi_{n}$. The sensor weights determine the total information and constrain the attainable imbalance, while, for fixed weights, the bearings determine the achieved directional balance.

### 3.3. Frame Potential and Tight-Frame Completion

Define the augmented collection of weighted information vectors as $\Phi_{n}=\Phi_{0}\cup \left\{\sqrt{w_{1}}b_{1},\sqrt{w_{2}}b_{2},\ldots ,\sqrt{w_{n}}b_{n}\right\}$. The frame operator associated with $\Phi_{n}$ has the matrix representation $J_{n}$. The frame potential of $\Phi_{n}$ is

(26) $$\mathrm{FP}\left(\Phi_{n}\right)=\sum_{u\in \Phi_{n}}\sum_{v\in \Phi_{n}}{\left|u^{T}v\right|}^{2}=\mathrm{tr}\left(J_{n}^{2}\right)=\lambda_{n,1}^{2}+\lambda_{n,2}^{2}=\frac{S_{n}^{2}+\rho_{n}^{2}}{2}.$$

For fixed sensor weights, $S_{n}$ does not depend on the additional sensor bearings. It follows from (26) that minimizing the frame potential is equivalent to minimizing $\rho_{n}$. Equation (24) shows that minimizing $\rho_{n}$ also minimizes the A-optimal cost. Therefore, the frame-potential and A-optimal criteria give the same optimal bearing configurations for the problem considered here.

When $\rho_{n}=0$, the augmented FIM has two equal eigenvalues and satisfies $J_{n}=\left(S_{n}/2\right)I_{2}$. In this case, the augmented weighted information vectors form a tight frame, the frame potential reaches $S_{n}^{2}/2$, and the A-optimal cost reaches $4/S_{n}$. The additional sensors are therefore said to complete the existing information frame into a tight frame.

From (22), tight-frame completion requires

(27) $$\sum_{j=1}^{n}w_{j}\exp\left(i\;2{\bar{\phi }}_{j}\right)=\kappa .$$

Thus, the vector sum of the additional double-angle vectors must reach the endpoint of the fixed imbalance vector κ on the positive real axis. Equivalently, the edge lengths $\kappa ,w_{1},w_{2},\ldots ,w_{n}$ must satisfy the polygon-closure condition.

A closed polygon exists if and only if

(28) $$2\max\left\{\kappa ,w_{1},w_{2},\ldots ,w_{n}\right\}\leq \kappa +\sum_{j=1}^{n}w_{j}.$$

This condition is the two-dimensional geometric form of the prescribed-norm tight-frame completion condition. It states that the longest edge cannot be longer than the sum of all the remaining edges. If it holds, the additional sensor bearings can be selected so that $\rho_{n}=0$. Otherwise, exact tight-frame completion is impossible, and a nonzero residual imbalance remains.

**Figure 3:**
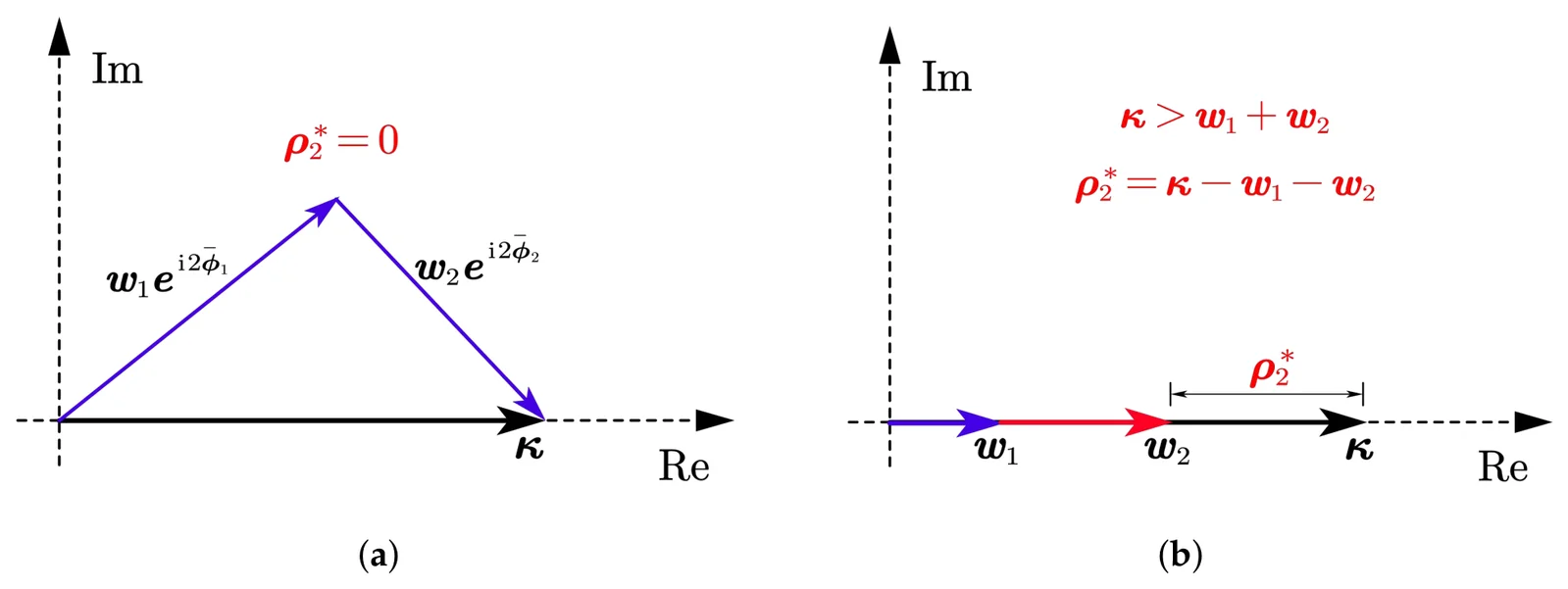
Polygon-closure interpretation for two additional sensors in the double-angle plane. (**a**) Closed case. (**b**) Non-closed collinear optimum.

For clarity, Figure 3 illustrates the two-sensor case in the double-angle plane. If the three lengths κ, $w_{1}$, and $w_{2}$ satisfy the triangle inequality, the added vectors can reach the endpoint of the fixed vector κ, giving $\rho_{2}^{★}=0$. If the triangle inequality is violated, the minimum residual is obtained by a collinear arrangement. For example, when $\kappa >w_{1}+w_{2}$, both added vectors are aligned with the positive real axis and $\rho_{2}^{★}=\kappa -w_{1}-w_{2}$. This example shows how the frame-theoretic analysis converts bearing design into a vector-balancing problem in the double-angle plane.

Tight-frame completion gives the best directional balance for the available total information, but it does not by itself guarantee satisfaction of the accuracy constraint. Even when $\rho_{n}=0$, the minimum cost $4/S_{n}$ may remain greater than ε if the total information is insufficient.

### 3.4. Optimal Performance for a Fixed Number of Additional Sensors

For a fixed number *n* of additional sensors with information weights $w_{1},\ldots ,w_{n}$, the total information $S_{n}$ is independent of their bearings. According to (24), minimizing the A-optimal cost is therefore equivalent to minimizing the residual imbalance $\rho_{n}$. The following theorem gives an explicit two-dimensional AOA formulation of the minimum residual imbalance and the corresponding A-optimal cost.

**Theorem** **1.** *For fixed edge lengths $\kappa ,w_{1},\ldots ,w_{n}$, define $L_{\max}=\max\left\{\kappa ,w_{1},\ldots ,w_{n}\right\}$, $L_{\mathrm{sum}}=\kappa +\sum_{j=1}^{n}w_{j}$. The minimum achievable residual imbalance is*

(29) $$\rho_{\min}\left(n\right)=\max\left\{0,\;2L_{\max}-L_{\mathrm{sum}}\right\}.$$

*The corresponding minimum A-optimal cost is*

(30) $$A_{\min}\left(n\right)=\frac{4S_{n}}{S_{n}^{2}-\rho_{\min}^{2}\left(n\right)},$$

*where $S_{n}$ is given by (20)*.

**Proof.**  For fixed sensor weights, $S_{n}$ is fixed. Equation (24) shows that the A-optimal cost is minimized when $\rho_{n}$ is minimized. The total length of all edges other than the longest one is $L_{\mathrm{sum}}-L_{\max}$. By the triangle inequality, these edges can cancel at most this amount of the longest edge. Therefore, $\rho_{n}\geq L_{\max}-\left(L_{\mathrm{sum}}-L_{\max}\right)=2L_{\max}-L_{\mathrm{sum}}$. Together with $\rho_{n}\geq 0$, this gives the lower bound in (29).

If $2L_{\max}\leq L_{\mathrm{sum}}$, the polygon closure condition in (28) holds, so the edges can form a closed polygon and $\rho_{n}=0$ is attainable. If $2L_{\max}>L_{\mathrm{sum}}$, equality is attained by directing all the remaining edges opposite to the longest edge. The resulting residual is the part of the longest edge that cannot be canceled. Substituting this minimum residual into (24) gives (30).    □

Theorem 1 gives the best performance attainable for any fixed number and set of positive sensor weights. Thus, $A_{\min}\left(n\right)$ can be evaluated without solving a multivariable bearing-optimization problem.

For equal-weight additional sensors, the result has a simpler form.

**Corollary** **1.** *Suppose that $w_{j}=w$ for all j. Then, $S_{n}=S_{0}+nw$ and*

(31) $$\rho_{\min}\left(n\right)=\left\{\begin{array}{cc} |\kappa -w|,\; & n=1,\\ \max\{0,\kappa -nw\},\; & n\geq 2. \end{array}\right.$$

**Proof.**  For n=1, the two edge lengths are κ and *w*, and the minimum magnitude of their vector sum is |κ−w|.

For n≥2, each added-sensor edge satisfies w≤κ+(n−1)w. Thus, an added-sensor edge cannot be longer than all the remaining edges combined. Only the original edge of length κ can prevent polygon closure. Applying Theorem 1 therefore gives $\rho_{\min}\left(n\right)=\max\left\{0,\kappa -nw\right\}$.    □

For one additional sensor, the minimum residual is attained by aligning its information direction with the weak information direction of the existing FIM. The same alignment is optimal for n≥2 when nw<κ, because the total additional information is insufficient to remove the original eigenvalue gap. The augmented FIM in the eigencoordinate system is then

(32) $${\bar{J}}_{n}=\left[\begin{array}{cc} \kappa_{1} & 0\\ 0 & \kappa_{2}+nw \end{array}\right],$$

and the corresponding minimum cost is

(33) $$A_{\min}\left(n\right)=\frac{1}{\kappa_{1}}+\frac{1}{\kappa_{2}+nw}.$$

Equation (33) applies to n=1 for any w>0, and to n≥2 when nw<κ. If n=1 and w>κ, the additional information exceeds the original eigenvalue gap and reverses the strong and weak directions. Nevertheless, alignment with the original weak direction remains optimal because a single rank-one information contribution cannot be divided between different directions.

When n≥2 and nw≥κ, the additional double-angle vectors can be arranged to cancel the original imbalance. In this case, $\rho_{\min}\left(n\right)=0$, the augmented FIM has equal eigenvalues, and

(34) $$A_{\min}\left(n\right)=\frac{4}{S_{0}+nw},\;n\geq 2,\;nw\geq \kappa .$$

A special case occurs when n=1 and w=κ. A single additional sensor then removes the original eigenvalue gap and produces a tight-frame FIM, giving $A_{\min}\left(1\right)=4/\left(S_{0}+w\right)$. If n=1 and w≠κ, exact tight-frame completion is impossible.

For n≥2, the equal-weight result contains two operating ranges. When nw<κ, the additional sensors compensate for the original weak information direction. When nw≥κ, their bearings can be jointly selected to produce a tight-frame FIM. These performance results are used in Section 4 to determine the minimum required number of sensors and construct the corresponding optimal bearings.

## 4. Minimum Sensor Augmentation and Geometry Optimization

Based on the performance results in Section 3, this section determines the minimum number of additional sensors required to satisfy the prescribed accuracy constraint. This threshold-based problem differs from determining the minimum number required solely for tight-frame completion because accuracy feasibility depends jointly on directional imbalance and total information. Optimal bearing constructions are then given for equal-weight sensors. The design is also extended to sensors with unequal information weights.

### 4.1. Determining the Minimum Number of Additional Sensors

If $A_{0}\leq \varepsilon$, the existing network already satisfies the accuracy constraint, and no additional sensors are required. Therefore, $n^{★}=0$. The following analysis considers the case $A_{0}>\varepsilon$.

#### 4.1.1. Single-Sensor Test

A single sensor provides a rank-one information contribution that cannot be divided between different directions. Its optimal information direction is therefore aligned with the weak information direction of the existing FIM. From (33), the best single-sensor cost is $A_{\min}\left(1\right)=1/\kappa_{1}+1/\left(\kappa_{2}+w\right)$. If $A_{\min}\left(1\right)\leq \varepsilon$, then $n^{★}=1$. When w=κ, the additional sensor exactly removes the eigenvalue gap and produces a tight-frame FIM. When w≠κ, the two eigenvalues remain unequal, but the accuracy constraint may still be satisfied.

If $A_{\min}\left(1\right)>\varepsilon$, at least two additional sensors are required. For n≥2, the equal-weight result in Section 3.4 can be written as

(35) $$A_{\min}\left(n\right)=\left\{\begin{array}{cc} \frac{1}{\kappa_{1}}+\frac{1}{\kappa_{2}+nw},\; & nw<\kappa ,\\ \frac{4}{S_{0}+nw},\; & nw\geq \kappa . \end{array}\right.\;n\geq 2.$$

The first case corresponds to compensation along the weak information direction. The second case applies when the added information is sufficient to produce a tight-frame FIM.

#### 4.1.2. Minimum Number of Additional Sensors in the Weak-Direction Stage

When n≥2 and nw<κ, the accuracy constraint becomes $1/\kappa_{1}+1/\left(\kappa_{2}+nw\right)\leq \varepsilon$. A solution in this stage can exist only if $\varepsilon >1/\kappa_{1}$. Under this condition, solving the inequality for *n* gives

(36) $$n_{\mathrm{weak}}=\max\left\{2,\;\left\lceil \frac{\frac{1}{\varepsilon -1/\kappa_{1}}-\kappa_{2}}{w}\right\rceil \right\}.$$

The value $n_{\mathrm{weak}}$ is valid only if it remains in the weak-direction stage; that is, if $n_{\mathrm{weak}}w<\kappa$. When this condition holds, $n^{★}=n_{\mathrm{weak}}$. If $\varepsilon \leq 1/\kappa_{1}$, or if $n_{\mathrm{weak}}w\geq \kappa$, the accuracy constraint cannot be met within this stage.

#### 4.1.3. Minimum Number of Additional Sensors in the Tight-Frame Stage

When n≥2 and nw≥κ, the additional sensor bearings can be selected so that the augmented FIM has equal eigenvalues. According to (34), the minimum A-optimal cost in this stage is $A_{\min}\left(n\right)=4/\left(S_{0}+nw\right)$.

The number of additional sensors must satisfy n≥2, nw≥κ, and $4/(S_{0}+nw)\leq \varepsilon$. The first condition ensures that at least two adjustable information vectors are available for the tight-frame construction. The second ensures that the additional information is sufficient to remove the original eigenvalue gap. The third ensures that the total information is sufficient to satisfy the prescribed accuracy constraint.

Solving these conditions and taking the smallest admissible integer gives

(37) $$n_{\mathrm{tight}}=\max\left\{2,\;\left\lceil \frac{\kappa }{w}\right\rceil ,\;\left\lceil \frac{4/\varepsilon -S_{0}}{w}\right\rceil \right\}.$$

The three terms in (37) correspond to the minimum number required for the multi-sensor construction, removal of the original eigenvalue gap, and satisfaction of the accuracy constraint, respectively.

Combining the existing-network test, the single-sensor test, and the minimum number of sensors in the two augmentation stages gives the following result.

**Theorem** **2.** *For equal-weight additional sensors with information weight w>0, the minimum number of additional sensors required to satisfy $A_{\min}\left(n\right)\leq \varepsilon$ is*

(38) $$n^{★}=\left\{\begin{array}{cc} 0,\; & A_{0}\leq \varepsilon ,\\ 1,\; & A_{0}>\varepsilon \;\mathrm{and}\;A_{\min}\left(1\right)\leq \varepsilon ,\\ n_{\mathrm{weak}},\; & A_{\min}\left(1\right)>\varepsilon ,\;\varepsilon >1/\kappa_{1},\;n_{\mathrm{weak}}w<\kappa ,\\ n_{\mathrm{tight}},\; & \mathrm{otherwise}, \end{array}\right.$$

*where $n_{\mathrm{weak}}$ and $n_{\mathrm{tight}}$ are given by (36) and (37), respectively, and $A_{\min}\left(1\right)$ follows from (33)*.

**Proof.**  If $A_{0}\leq \varepsilon$, the existing network already satisfies the accuracy constraint, and hence $n^{★}=0$. If $A_{0}>\varepsilon$ but $A_{\min}\left(1\right)\leq \varepsilon$, one additional sensor is both necessary and sufficient, giving $n^{★}=1$.

Suppose that $A_{\min}\left(1\right)>\varepsilon$. In the weak-direction stage, the cost given by (33) decreases strictly as *n* increases. Solving $A_{\min}\left(n\right)\leq \varepsilon$ gives $n_{\mathrm{weak}}$. This value belongs to the weak-direction stage only when $\varepsilon >1/\kappa_{1}$ and $n_{\mathrm{weak}}w<\kappa$.

If these conditions are not satisfied, the minimum feasible number of sensors must lie in the tight-frame stage. In this stage, the requirements n≥2, nw≥κ, and $4/(S_{0}+nw)\leq \varepsilon$ give the three integer lower bounds in (37). Their maximum is therefore the smallest feasible value in this stage. Combining these cases gives (38).    □

Theorem 2 requires only the eigendecomposition of the 2×2 existing FIM, followed by scalar calculations and ceiling operations. It avoids solving a separate bearing-optimization problem for every possible number of sensors.

For numerical implementation, $n^{★}$ can also be found using a sequential closed-form test. Starting from n=0, evaluate $A_{\min}\left(n\right)$ using the single-sensor expression and (35). The procedure stops at the first *n* satisfying $A_{\min}\left(n\right)\leq \varepsilon$. This alternative is useful near the boundary between the weak-direction and tight-frame stages and still requires only scalar calculations.

### 4.2. Optimal Bearing Constructions for Equal-Weight Sensors

After $n^{★}$ has been determined, the additional sensor bearings must be selected so that $A_{\min}\left(n^{★}\right)$ is attained. Recall that ${\bar{\phi }}_{j}=\phi_{j}-\vartheta$ denotes the bearing of the *j*-th additional sensor relative to the strong information direction of the existing FIM. In the double-angle plane, the corresponding information vector has direction $2{\bar{\phi }}_{j}$. The construction is therefore first carried out in the double-angle plane, after which the resulting angles are divided by two to obtain the relative sensor bearings.

#### 4.2.1. Weak-Direction Compensation

When n=1, or when n≥2 and nw<κ, the minimum cost is attained by aligning the additional information with the weak information direction of the existing FIM. The corresponding relative sensor bearings are ${\bar{\phi }}_{j}=k_{j}\pi$, $k_{j}\in Z$, j=1,2,…,n.

For these bearings, the transformed information-direction vector is ${\bar{b}}_{j}={\left[0,-1\right]}^{T}$, up to a possible sign change. Its outer product therefore adds information only to the original weak direction. The resulting augmented FIM and minimum A-optimal cost are given by (32) and (33), respectively.

#### 4.2.2. Tight-Frame Construction

When n≥2 and nw≥κ, the additional sensor bearings can be selected so that the augmented FIM has equal eigenvalues. From (27), the relative bearings must satisfy

(39) $$\sum_{j=1}^{n}\exp\left(i2{\bar{\phi }}_{j}\right)=\kappa /w.$$

The following constructions are specified in terms of information directions. They provide bearing configurations that attain the analytical minimum residual imbalance. Physical deployment constraints, such as minimum inter-sensor separation or prohibition of collocated sensors, are treated as additional implementation constraints and are discussed after the construction.

**Proposition** **1.** *Suppose that n≥2 and nw≥κ.*

1. *If n is even, an optimal set of relative bearings is*
(40) $$\begin{array}{ccc} {\bar{\phi }}_{j}\; & =\left\{\begin{array}{cc} +\frac{\alpha_{e}}{2},\; & j=1,2,\ldots ,\frac{n}{2}\\ -\frac{\alpha_{e}}{2},\; & j=\frac{n}{2}+1,\ldots ,n \end{array}\right.,\;\alpha_{e}\; & =\arccos\left(\frac{\kappa }{nw}\right). \end{array}$$
2. *If n≥3 is odd, an optimal set of relative bearings is*
(41) $$\begin{array}{cc} {\bar{\phi }}_{j}\; & =\left\{\begin{array}{cc} 0,\; & j=1\\ +\frac{\alpha_{o}}{2},\; & j=2,\ldots ,\frac{n+1}{2}\\ -\frac{\alpha_{o}}{2},\; & j=\frac{n+3}{2},\ldots ,n \end{array}\right.,\;\alpha_{o}=\arccos\left(\frac{\kappa /w-1}{n-1}\right). \end{array}$$

*Both constructions satisfy (39). Consequently, they give $\rho_{n}=0$, and the augmented FIM has two equal eigenvalues.*

**Proof.**  For even *n*, the construction in (40) places n/2 double-angle vectors at $+\alpha_{e}$ and n/2 vectors at $-\alpha_{e}$. Their vertical components cancel, and

(42) $$\sum_{j=1}^{n}\exp\left(i\;2{\bar{\phi }}_{j}\right)=\frac{n}{2}\exp\left(i\alpha_{e}\right)+\frac{n}{2}\exp\left(-i\alpha_{e}\right)=n\cos\alpha_{e}=\frac{\kappa }{w}.$$

Thus, (39) holds.

For odd *n*, one double-angle vector is placed at zero, while the remaining n−1 vectors form symmetric pairs at $+\alpha_{o}$ and $-\alpha_{o}$. Therefore,

(43) $$\sum_{j=1}^{n}\exp\left(i\;2{\bar{\phi }}_{j}\right)=1+\frac{n-1}{2}\exp\left(i\alpha_{o}\right)+\frac{n-1}{2}\exp\left(-i\alpha_{o}\right)=\frac{\kappa }{w}.$$

Hence, the odd-number construction also satisfies (39). In both cases, the residual imbalance is zero, so the augmented FIM has equal eigenvalues and attains the minimum A-optimal cost.    □

**Remark** **1.** *Proposition 1 provides optimal bearing constructions without minimum inter-sensor separation constraints. Under a common-radius deployment, the π-ambiguity provides at most two distinct physical positions for each information direction, so repeated bearings may lead to collocation, especially for large n. If different deployment ranges are used to avoid collocation, the resulting information weights must be preserved or the unequal-weight design must be recomputed. Under strict common-radius and minimum-separation constraints, the analytical construction should be interpreted as a performance lower bound and an initialization for constrained placement refinement.*

For both constructions, $J_{n}=\left[\left(S_{0}+nw\right)/2\right]I_{2}$ and $A_{\min}\left(n\right)=4/\left(S_{0}+nw\right)$. When nw=κ, both constructions give $\alpha_{e}=\alpha_{o}=0$. All additional information is then placed along the original weak information direction and exactly removes the eigenvalue gap. When nw>κ, the additional information must be distributed across multiple directions to prevent overcompensation and maintain equal FIM eigenvalues.

#### 4.2.3. Global Bearings and Sensor Positions

After the relative bearings have been obtained, the global bearing of the *j*-th additional sensor satisfies $\phi_{j}\equiv \vartheta +{\bar{\phi }}_{j}\;\left(\mathrm{mod}\;\pi \right)$. This relation means that the information design determines the bearing only up to an integer multiple of π. Indeed, it follows from (12) that $b\left(\phi_{j}+\pi \right)=-b\left(\phi_{j}\right)$, so replacing $\phi_{j}$ with $\phi_{j}+\pi$ does not change the outer product $b_{j}b_{j}^{T}$ or the augmented FIM.

A representative bearing can first be selected in [0,π). Either this bearing or its opposite direction, obtained by adding π, can then be used in the physical range [0,2π). This freedom allows sensors with the same information direction to be placed on opposite sides of the target, which can reduce physical overlap without changing localization performance.

For additional sensors deployed at a common target distance $r_{a}$, their physical positions are obtained from (13) using the selected global bearings.

The constructions in Proposition 1 provide simple symmetric solutions, but the optimal bearing configuration is generally not unique. Any set of relative bearings satisfying (39) produces the same balanced FIM and A-optimal cost. If boundaries, obstacles, or minimum-separation requirements are present, another solution satisfying the same tight-frame condition may be selected when such a feasible configuration exists.

### 4.3. Extension to Unequal Information Weights

For sensors with unequal weights, the best achievable cost is already given by (29) and (30). Therefore, the total information and polygon quantities do not need to be derived again.

For a fixed set of weights $w_{1},\ldots ,w_{n}$, tight-frame completion is possible when the polygon condition (28) holds. The weighted double-angle vectors can then be arranged to form a closed polygon. If the condition does not hold, the minimum-residual arrangement described after (29) is used.

Let $\psi_{j}$ denote the direction of the *j*-th additional information vector in the double-angle plane. The corresponding relative and global bearings are ${\bar{\phi }}_{j}=\psi_{j}/2$ and $\phi_{j}\equiv \vartheta +{\bar{\phi }}_{j}\;\left(\mathrm{mod}\;\pi \right)$, respectively.

If unequal-weight sensors are added in a fixed order, the minimum number can be found by evaluating (30) after each sensor is added and stopping when the accuracy constraint is satisfied. If sensors must instead be selected from a larger set of available sensors with different weights or costs, sensor selection is also required. In that case, (30) can be used to evaluate the best possible performance of each possible subset.

### 4.4. Computational Procedure

The main matrix calculation is the eigendecomposition of the 2×2 existing FIM. For equal-weight sensors, the remaining steps use scalar expressions, ceiling operations, and direct angle calculations, so no multivariable bearing optimization is required. For unequal-weight sensors, the analytical minimum cost is used to determine $n^{★}$, after which a weighted angular minimization is performed to construct the corresponding bearings. When collocation or minimum-separation constraints are imposed, the constructed bearings are used as an initialization for a constrained deployment refinement.

The minimum-residual weighted arrangement in Algorithm 1 is computed as follows. Let $\ell_{0}=\kappa ,\ell_{1}=w_{1},\ldots ,\ell_{n}=w_{n}$, where $\ell_{0}$ represents the existing imbalance edge. We fix its direction as $\alpha_{0}=\pi$ and compute the remaining edge angles by minimizing ${\left|\ell_{0}e^{i\alpha_{0}}+\sum_{j=1}^{n}\ell_{j}e^{i\alpha_{j}}\right|}^{2}$ over $\alpha_{1},\ldots ,\alpha_{n}$. When the polygon-closure condition in Equation (28) holds, the global minimum is zero and the resulting angles form a closed weighted polygon. When the condition does not hold, the minimum residual is $2\ell_{\max}-\sum_{j=0}^{n}\ell_{j}$, consistent with Theorem 1. The added-sensor bearings are then recovered from ${\bar{\phi }}_{j}=\alpha_{j}/2$ modulo π.

For the unequal-weight construction, the angular minimization is implemented in MATLAB R2024a using fminsearch with 32 initial angle configurations. The first two configurations consist of zero angles and uniformly distributed double-angle directions, respectively, while the remaining configurations are independently sampled from [0,2π). The optimization settings are MaxIter=3500, MaxFunEvals=25,000, $\mathrm{TolX}=10^{-10}$, and $\mathrm{TolFun}=10^{-12}$. The solution with the smallest residual is retained, and polygon closure is accepted when the residual magnitude is below $10^{-9}$.
**Algorithm 1** AOA network augmentation procedure**Require:** Existing network, nominal target position p, accuracy threshold ε, and added-sensor parameters
**Ensure:** Minimum number of additional sensors $n^{★}$, optimal bearings $\phi^{★}$, and sensor positions  1: Compute the existing FIM $J_{0}$  2: Obtain $\kappa_{1}$, $\kappa_{2}$, and ϑ from the eigendecomposition of $J_{0}$  3: Compute $S_{0}=\kappa_{1}+\kappa_{2}$, $\kappa =\kappa_{1}-\kappa_{2}$, and $A_{0}=1/\kappa_{1}+1/\kappa_{2}$  4: **if** 
$A_{0}\leq \varepsilon$ 
**then**  5:        **return** $n^{★}=0$ and $\phi^{★}=⌀$  6: **end if**  7: Compute the added-sensor information weights  8: **if** the additional sensors have equal weights **then**  9:        Determine $n^{★}$ using Theorem 210:        **if** $n^{★}=1$ or $n^{★}w<\kappa$ **then**11:               Use the weak-direction construction12:        **else**13:               Use the even- or odd-number tight-frame construction14:        **end if**15: **else**16:        Determine $n^{★}$ sequentially using (30)17:        Construct a minimum-residual weighted arrangement by weighted angular minimization18: **end if**19: Convert the relative bearings into global bearings $\phi^{★}$20: Compute the corresponding sensor positions21: Verify $\mathrm{tr}(J_{n^{★}}^{-1})\leq \varepsilon$22: **return** $n^{★}$, $\phi^{★}$, and the sensor positions

## 5. Simulation Results

This section validates the analytical results and compares the proposed design with several reference geometries. Further experiments examine unequal information weights, random existing networks, and target-position mismatch. Deterministic results are computed from the closed-form expressions, whereas Monte Carlo results are evaluated using independent random-seed batches to obtain confidence intervals.

### 5.1. Simulation Setup

A two-dimensional AOA localization network is considered. The nominal target is located at $p={\left[0,0\right]}^{T}m$, and the existing network contains four fixed sensors. Their bearings, target distances, and Cartesian coordinates are listed in Table 1.

The simulated sensor data are noisy AOA bearing measurements generated from the true target position and known sensor positions. Unless otherwise stated, all AOA errors are mutually independent zero-mean Gaussian variables with a standard deviation of $2^{\circ }$ for both existing and additional sensors. Angular standard deviations are converted to radians when calculating the information weights, FIMs, and measurement noise samples. No missing measurements, outliers, or deliberately corrupted data points are included in the baseline simulations.

The FIM of the existing network is

(44) $$J_{0}=\left[\begin{array}{cc} 0.00460499 & -0.00503677\\ -0.00503677 & 0.02531160 \end{array}\right]\;m^{-2}.$$

Its eigenvalues are $\kappa_{1}=2.6471767\times 10^{-2}\;m^{-2}$ and $\kappa_{2}=3.4448275\times 10^{-3}\;m^{-2}$. The resulting information parameters are $S_{0}=2.9916594\times 10^{-2}\;m^{-2}$, $\kappa =2.3026939\times 10^{-2}\;m^{-2}$, and $\vartheta =102.971^{\circ }$.

The original A-optimal cost is $A_{0}=328.0664\;m^{2}$, corresponding to an equivalent CRLB RMSE of 18.1126m. The FIM condition number is 7.6845, indicating a strongly unbalanced information distribution. The existing network therefore does not satisfy the prescribed threshold $\varepsilon =60\;m^{2}$, whose equivalent CRLB RMSE threshold is $\sqrt{\varepsilon }=7.746\;m$.

Each equal-weight additional sensor provides an information weight of $w=9.1189065\times 10^{-3}\;m^{-2}$, giving κ/w=2.5252. The theoretical performance is evaluated using the CRLB trace, equivalent CRLB RMSE, FIM condition number, and normalized information imbalance.

The empirical localization performance is evaluated using 10,000 Monte Carlo trials and an iterative weighted nonlinear least-squares estimator. The trials are divided into 20 independent batches with 500 trials per batch. Different random seeds are used for different batches, and 95% confidence intervals are computed from the batch-wise empirical metrics using Student’s *t* distribution.

(45) $$\hat{p}=\underset{p}{\arg\min}\sum_{i=1}^{m+n}\frac{{\mathrm{warp}}^{2}\left[{\tilde{\theta }}_{i}-h_{i}\left(p\right)\right]}{\sigma_{i}^{2}},$$

where warp(·) maps an angular residual to [−π,π). The minimization in (45) is solved by iterative weighted least squares. Let $r^{\left(k\right)}$ be the vector of wrapped bearing residuals and let $G^{\left(k\right)}$ be the Jacobian matrix of the predicted bearings evaluated at $p^{\left(k\right)}$. With $W=\mathrm{diag}(1/\sigma_{1}^{2},\ldots ,1/\sigma_{m+n}^{2})$, the update is

(46) $$p^{\left(k+1\right)}=p^{\left(k\right)}+{\left[{\left(G^{\left(k\right)}\right)}^{T}WG^{\left(k\right)}+10^{-10}I\right]}^{-1}{\left(G^{\left(k\right)}\right)}^{T}Wr^{\left(k\right)}.$$

The initial estimate is fixed as ${\left[15,-12\right]}^{T}\;m$ for all compared methods. The iteration is stopped when the update norm is smaller than $10^{-7}\;m$ or when the maximum number of 18 iterations is reached. The update norm is clipped to 60m to avoid unrealistically large steps. The same standardized noise samples are used for all compared methods to reduce Monte Carlo sampling differences.

The empirical localization RMSE is

(47) $${\mathrm{RMSE}}_{\mathrm{MC}}=\sqrt{\frac{1}{N_{\mathrm{MC}}}\sum_{\ell =1}^{N_{\mathrm{MC}}}{∥{\hat{p}}^{\left(\ell \right)}-p∥}_{2}^{2}}\;,\;N_{\mathrm{MC}}=10000.$$

### 5.2. Analytical Validation and Required Number of Additional Sensors

The number of equal-weight additional sensors is varied from n=0 to n=8. For each *n*, the analytical minimum A-optimal cost is evaluated using the results in Section 3. Independent multistart numerical optimization is also performed for n=1,…,5.

For n=1,…,5, the numerical solutions agree with the analytical minimum costs to machine precision, with relative differences on the order of $10^{-16}$. This agreement verifies the derived minimum residual imbalance and A-optimal cost.

**Figure 4:**
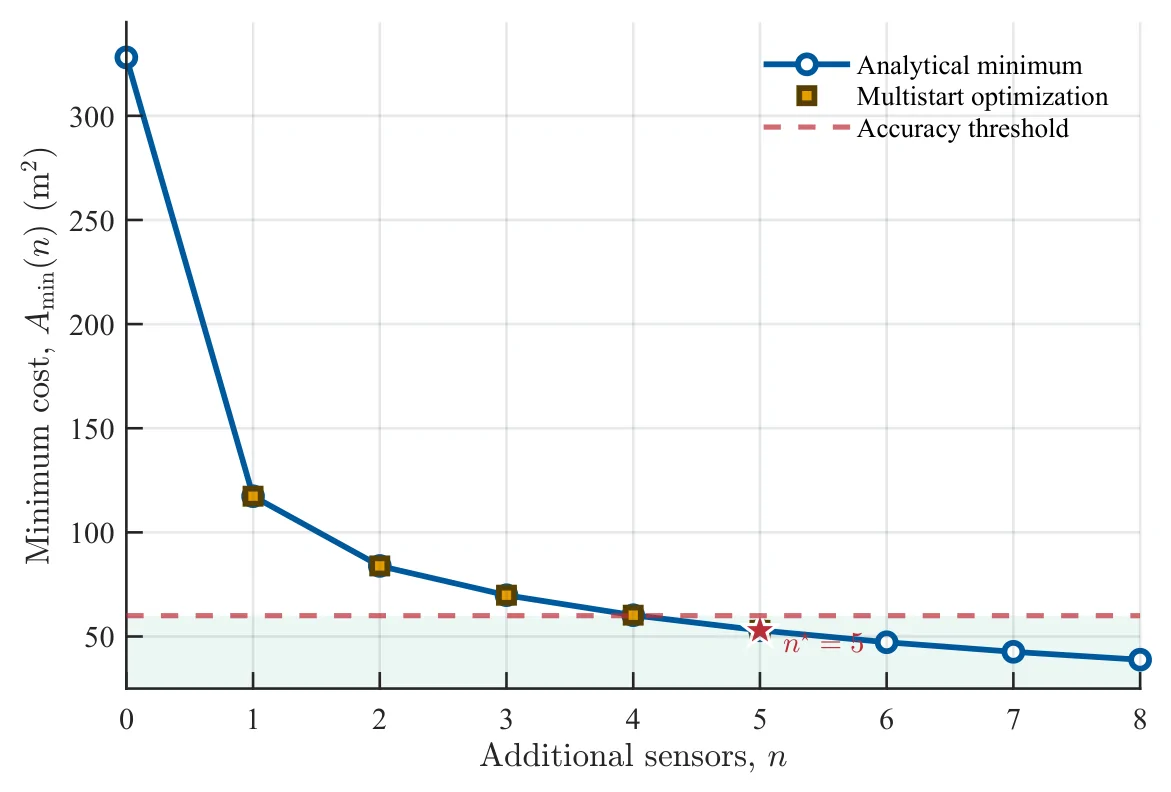
Minimum A-optimal cost versus the number of additional sensors. Green shading indicates $A_{\min}\left(n\right)\leq \varepsilon$; the red star marks $n^{★}=5$.

Figure 4 shows the minimum A-optimal cost as the number of additional sensors increases. The first two sensors operate in the weak-direction compensation stage because nw<κ. Since κ/w=2.5252, tight-frame completion becomes possible when n≥3. However, the minimum costs for n=3 and n=4 remain above the prescribed threshold. The smallest feasible number of sensors is therefore $n^{★}=5$, for which $A_{\min}\left(n^{★}\right)=52.9723\;m^{2}$.

Because $n^{★}=5$ is odd, the odd-number tight-frame construction is used. The double-angle offset is $\alpha =67.586^{\circ }$, corresponding to a physical bearing offset of $\alpha /2=33.793^{\circ }$. Combining this offset with $\vartheta =102.971^{\circ }$, and using the π-periodicity of the AOA information matrix to avoid physical overlap, gives

(48) $$\phi^{★}={\left[102.971^{\circ },136.764^{\circ },316.764^{\circ },69.178^{\circ },249.178^{\circ }\right]}^{T}.$$

**Figure 5:**
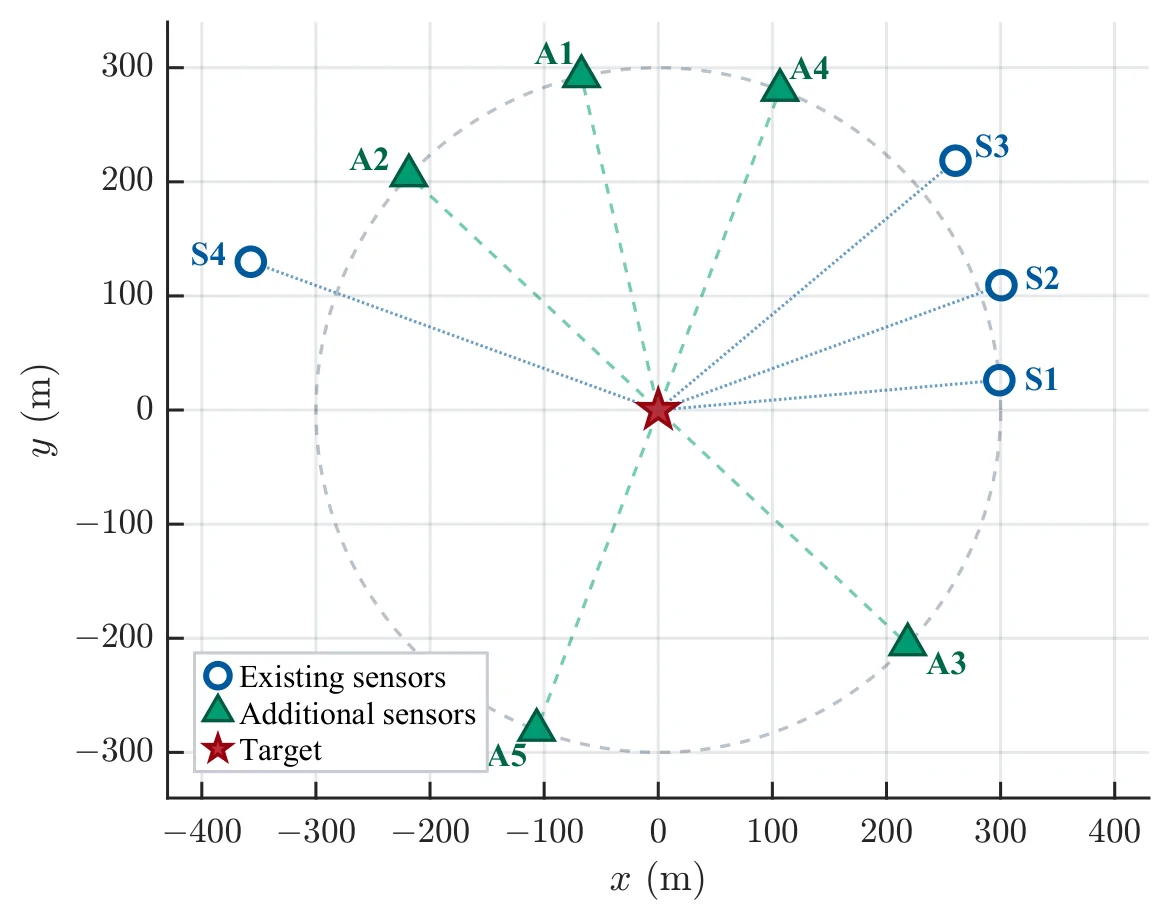
Existing sensors and the optimal augmentation geometry.

The resulting sensor geometry is shown in Figure 5. The two eigenvalues of the augmented FIM are both approximately $3.7756\times 10^{-2}\;m^{-2}$. Consequently, the augmented FIM has a condition number of one and a normalized imbalance of zero, confirming exact tight-frame completion.

### 5.3. Effects of the Accuracy Threshold and Sensor Quality

**Figure 6:**
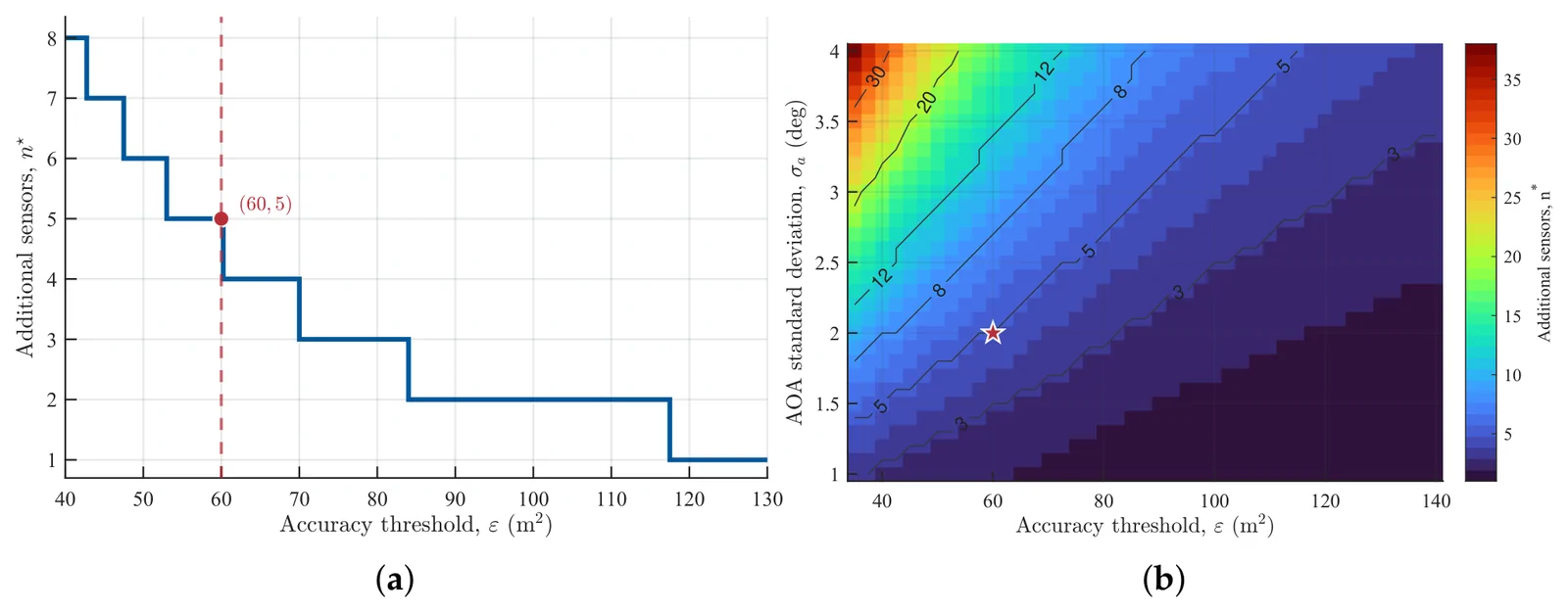
Required number $n^{★}$ of additional sensors. (**a**) Variation with the accuracy threshold. (**b**) Joint variation with the accuracy threshold and additional-sensor AOA uncertainty. The red star in (**b**) marks the nominal design point $\left(\varepsilon ,\sigma_{a}\right)=\left(60\;m^{2},2^{\circ }\right)$.

Figure 6a shows the required number of additional sensors as the accuracy threshold varies from 40 to $130\;m^{2}$. As ε decreases, the accuracy requirement becomes more stringent and the required number of additional sensors increases in a stepwise manner. For example, thresholds of 120, 80, 60, and $40\;m^{2}$ require 1, 3, 5, and 8 additional sensors, respectively. This staircase pattern is caused by the integer-valued number of sensors and the fixed information contribution of each additional sensor.

Figure 6b jointly varies the accuracy threshold and the AOA standard deviation of the additional sensors. The threshold ranges from 35 to $140\;m^{2}$, while the AOA standard deviation ranges from $1^{\circ }$ to $4^{\circ }$. A larger AOA uncertainty reduces the information weight *w* and therefore increases the required number of sensors. The nominal design point $\left(\varepsilon ,\sigma_{a}\right)=\left(60\;m^{2},2^{\circ }\right)$ lies in the region corresponding to $n^{★}=5$. It should be noted that the large-$n^{★}$ regions in Figure 6b represent the analytical minimum under the bearing-only design model. When $n^{★}$ is large, the explicit symmetric construction may assign multiple sensors to the same bearing. If a common deployment radius and a no-collocation constraint are both imposed, these regions should be interpreted as analytical lower bounds, and an additional separation-constrained placement refinement is required for physical deployment.

### 5.4. Comparison of Augmentation Geometries

Four augmentation geometries are compared using the same number of additional sensors, target distance, and AOA accuracy:

1. Proposed frame completion. The bearings are constructed using the strong information direction and eigenvalue gap of the existing FIM.
2. Uniform augmentation. The additional sensors are uniformly spaced over the full physical bearing-angle range φ∈[0,2π), namely $\varphi_{j}^{\mathrm{uni}}=2\pi \left(j-1\right)/n$, j=1,2,…,n. For n=5, the bearings are $0^{\circ }$, $72^{\circ }$, $144^{\circ }$, $216^{\circ }$, and $288^{\circ }$.
3. Weak-direction concentration. All additional information directions are aligned with the weak information direction of the existing network.
4. Fixed random configuration. A reproducible configuration with bearings $8.998^{\circ }$, $76.641^{\circ }$, $122.968^{\circ }$, $141.523^{\circ }$, and $4.637^{\circ }$.

The theoretical results are summarized in Table 2. All configurations contain the same amount of additional information. Their performance differences therefore arise from the directional distribution of this information.

Relative to uniform augmentation, weak-direction concentration, and the fixed random configuration, the proposed method reduces the theoretical CRLB trace by 9.30%, 8.93%, and 14.35%, respectively. Uniform augmentation balances the information supplied by the new sensors but does not directly compensate for the original information imbalance. Weak-direction concentration is optimal only before tight-frame completion becomes feasible. In the present experiment, 5w>κ; therefore, placing all additional information along the original weak direction causes overcompensation and reverses the eigenvalue imbalance.

Monte Carlo localization is performed for the proposed method, uniform augmentation, and the fixed random configuration. The theoretical equivalent CRLB RMSE, empirical RMSE, and empirical 95% error quantile are reported in Table 3. Values in brackets are 95% confidence intervals (CIs) computed from 20 independent Monte Carlo batches.

Across the 10,000 Monte Carlo trials conducted for each compared method, all trials satisfied the update-norm stopping criterion within 18 iterations, and no non-finite numerical failure was observed.

Relative to uniform augmentation and the fixed random configuration, the proposed method reduces the Monte Carlo RMSE by approximately 5.69% and 8.23%, respectively. It also reduces the empirical 95% error quantile by approximately 5.73% and 9.17%, respectively.

**Figure 7:**
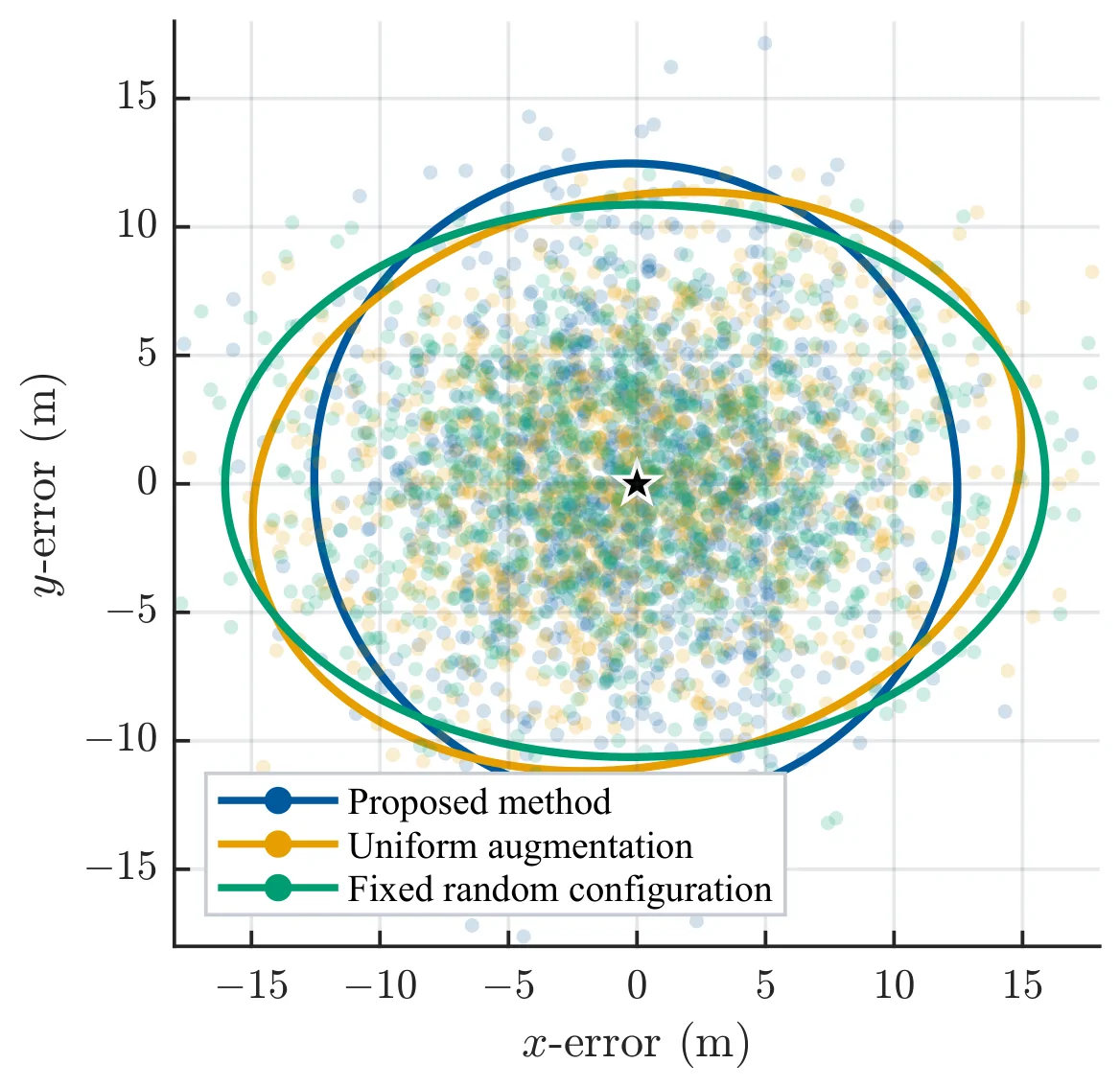
Monte Carlo localization errors and covariance ellipses with nominal 95% Gaussian coverage.

Figure 7 shows the Monte Carlo localization-error samples and covariance ellipses scaled to the nominal 95% Gaussian coverage. The blue, orange, and green curves correspond to the proposed method, uniform augmentation, and fixed random configuration, respectively, while the black star at the origin marks the zero-error reference. The covariance ellipse of the proposed method is closer to circular, consistent with the unit FIM condition number and zero information imbalance.

**Figure 8:**
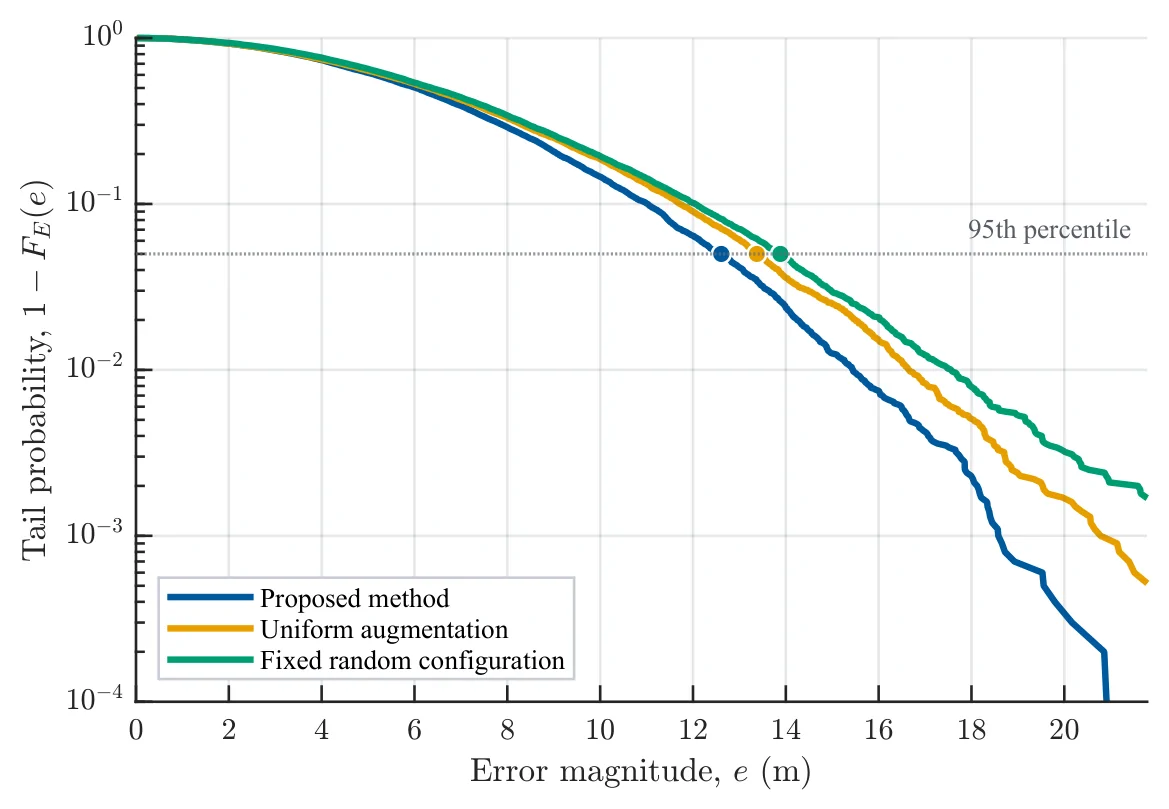
Tail probabilities of the localization errors.

Figure 8 shows the tail probability $1-F_{E}\left(e\right)=\mathrm{Pr}\left\{E>e\right\}$, where $E={∥\hat{p}-p∥}_{2}$. The three distributions are close in the central region, which is consistent with their equal number of sensors and equal amounts of additional information. Their differences become clearer in the large-error region, where the proposed method generally exhibits a lower tail probability.

### 5.5. Validation for Unequal Information Weights

The unequal-weight experiment examines the generalized polygon result when the additional sensors have different AOA accuracies and target distances. Four sensors are added in a prescribed order. Their AOA standard deviations are $1.2^{\circ }$, $2.4^{\circ }$, $2.8^{\circ }$, and $1.8^{\circ }$, and their target distances are 260, 320, 340, and 280m, respectively.

For n=1,2,3, and 4, the analytical minimum A-optimal costs are 64.6805, 58.1179, 54.9473, and $46.6462\;m^{2}$, respectively. The corresponding minimum residual magnitudes are $1.070\times 10^{-2}$, $5.131\times 10^{-3}$, $1.509\times 10^{-3}$, and $0\;m^{-2}$. Multistart numerical optimization reproduces all four analytical costs at the displayed precision.

**Figure 9:**
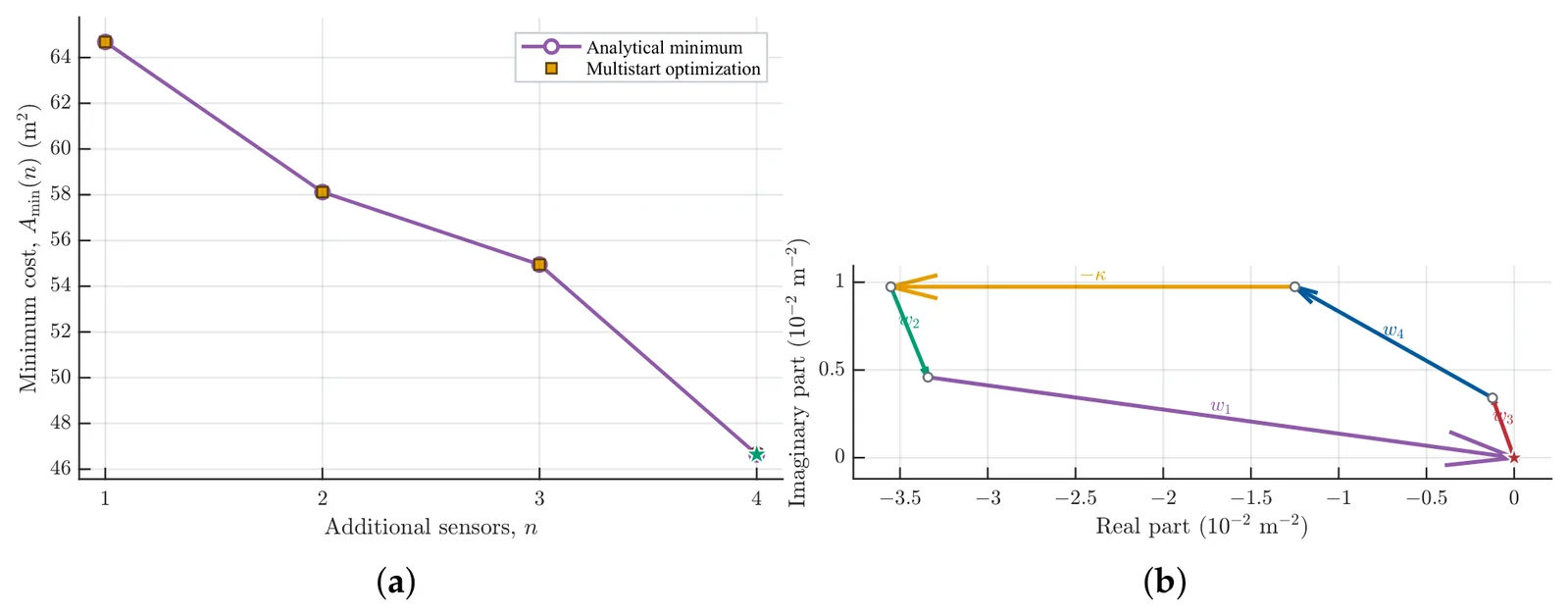
Validation for unequal information weights. (**a**) Comparison of the analytical and numerical minimum costs (green star: exact closure at n=4). (**b**) Weighted polygon closure after all four additional sensors are included (red star: common starting and closing point).

Figure 9a compares the analytical and numerical minimum costs. Exact polygon closure is not possible for the first three sensor sets. After the fourth sensor is added, the closure condition is satisfied, and the numerical residual decreases to $7.259\times 10^{-10}\;m^{-2}$, which is effectively zero within the optimization tolerance.

After the second sensor is added, $A_{\min}\left(2\right)=58.1179\;m^{2}<\varepsilon$, even though the polygon has not yet closed. This result confirms that tight-frame completion gives the best directional balance for a prescribed amount of information but is not necessary for satisfying an accuracy threshold.

Figure 9b shows the polygon obtained after all four sensors are included. This diagram is an auxiliary representation in the double-angle plane rather than a physical sensor-position plot. The fixed edge −κ and the four weighted information edges close the polygon within numerical tolerance, consistent with the generalized tight-frame condition.

### 5.6. Generalization Across Random Existing Networks

To examine whether the conclusions depend on a single existing geometry, 500 random networks are generated. Each network contains four sensors based on the nominal bearing pattern $\left[5^{\circ },20^{\circ },40^{\circ },160^{\circ }\right]$. A common rotation is uniformly sampled from [0,π), and independent zero-mean Gaussian bearing perturbations with a standard deviation of $12^{\circ }$ are added. The sensor distances are uniformly sampled from 260 to 420m, while their AOA standard deviations are uniformly sampled from $1.6^{\circ }$ to $2.4^{\circ }$.

**Figure 10:**
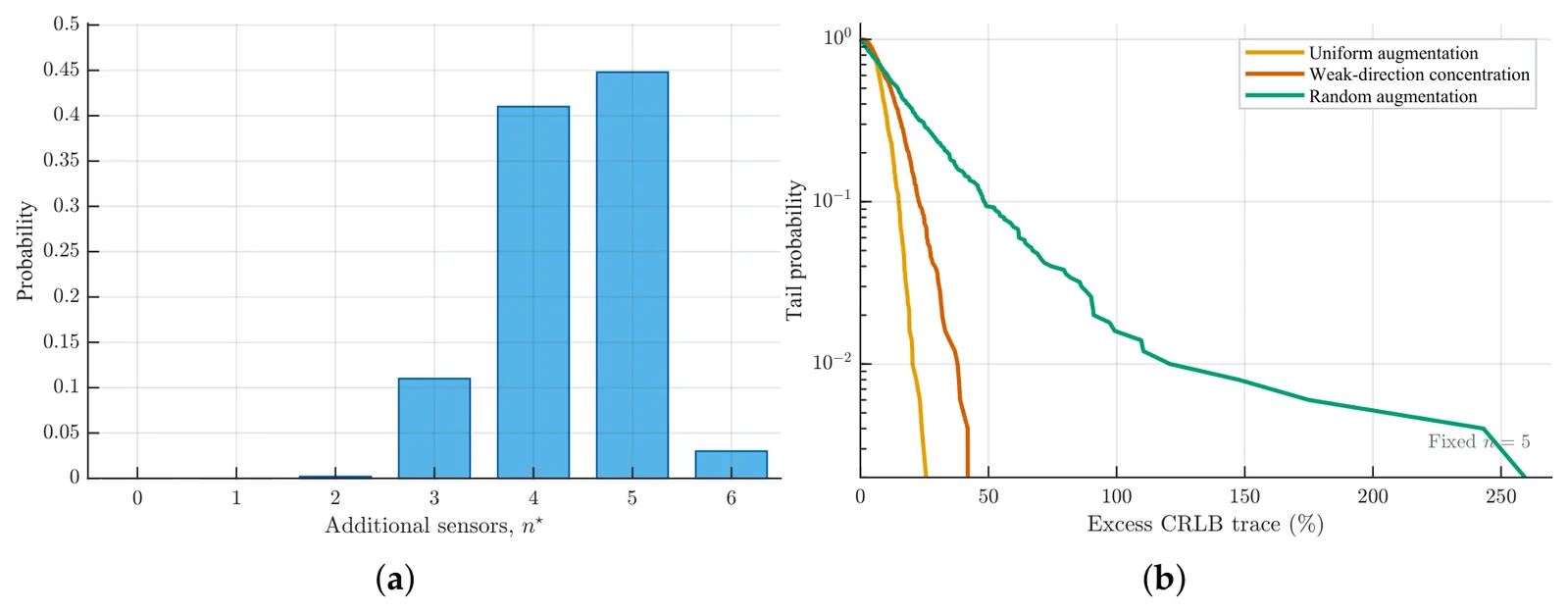
Results for random existing networks. (**a**) Distribution of the required number of additional sensors. (**b**) Tail probabilities of the excess CRLB trace for n=5.

For each network, the minimum number of additional sensors satisfying $\varepsilon =60\;m^{2}$ is calculated analytically. Figure 10a shows that the required numbers are mainly concentrated at four and five sensors. Smaller and larger values also occur because the existing networks differ in total information and directional imbalance.

For the geometry comparison in Figure 10b, the number of additional sensors is fixed at n=5. The excess cost of a reference method is defined as

(49) $$\Delta_{A}=100\left[\frac{A_{\mathrm{method}}}{A_{\min}\left(5\right)}-1\right]\%.$$

The random augmentation bearings are independently sampled from [0,π).

Uniform augmentation has a median excess CRLB trace of 8.22% and a 90th percentile excess of 14.90%. The corresponding values for weak-direction concentration are 11.76% and 22.66%, while those for random augmentation are 14.58% and 48.13%. The longer tail of the random configuration shows that uncoordinated bearing selection can sometimes produce severe directional imbalance.

Multistart numerical optimization is also performed for 20 randomly generated network cases, with the tested number of additional sensors cycling from one to five. The maximum relative difference between the analytical and numerical minimum costs is $4.783\times 10^{-16}$, confirming agreement to machine precision.

### 5.7. Sensitivity to Target-Position Mismatch

The proposed design is local and is constructed at the nominal target position. A final experiment therefore evaluates its sensitivity when the actual target differs from the nominal point while all deployed sensor positions remain fixed.

The target-offset radius is varied from 0 to 100m in increments of 10m. For each radius, 72 uniformly spaced offset directions are examined. At each actual target position and for each deployment, the achieved A-optimal cost is compared with the local information-theoretic minimum obtained by reorienting the additional information vectors while keeping the existing-network FIM and that deployment’s actual additional-sensor weights fixed. The resulting A-optimality gap is

(50) $$G_{A}=100\left(\frac{A_{\mathrm{deployed}}}{A_{\mathrm{local}\;\mathrm{optimum}}}-1\right)\%.$$

**Figure 11:**
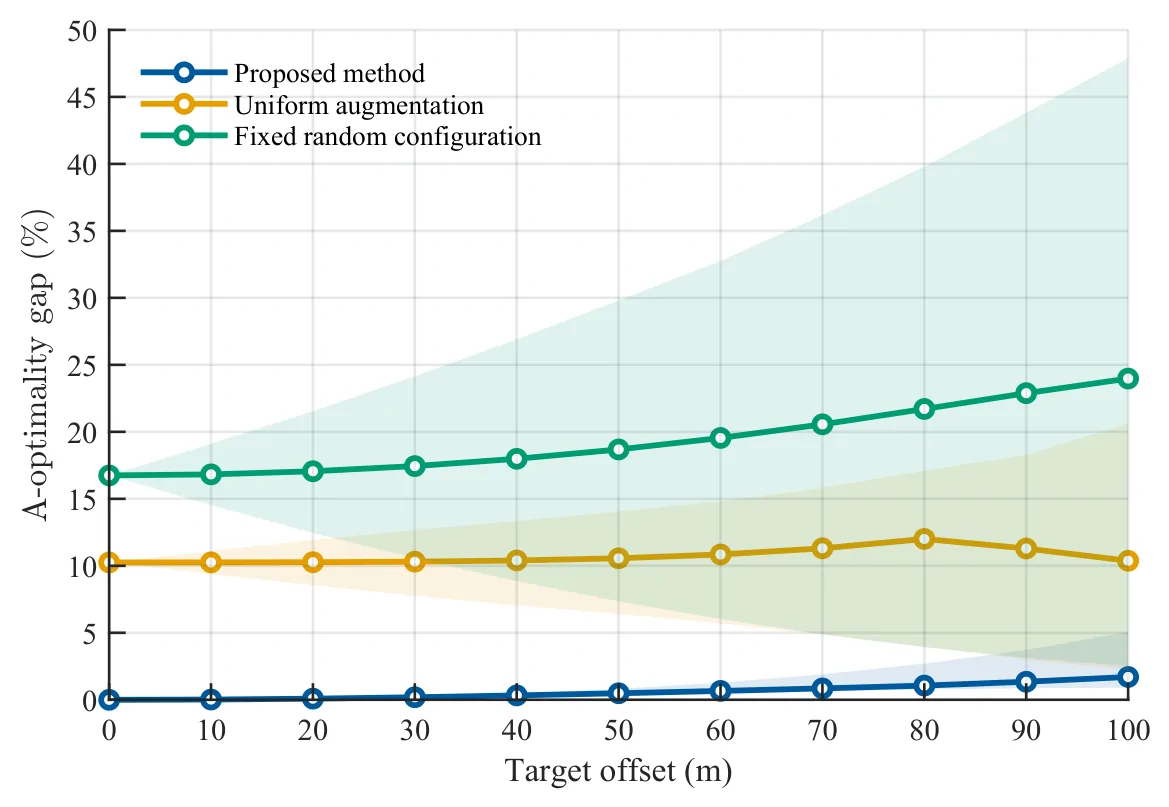
A-optimality gaps under target-position mismatch.

Figure 11 shows the median gaps as solid curves and the corresponding 10th–90th percentile intervals over the offset directions as colored shaded regions. At the nominal point, the proposed configuration has zero gap because it is the analytical optimum. Uniform augmentation and the fixed random configuration have nonzero gaps at zero offset because they are already suboptimal at the nominal point. Therefore, $G_{A}$ measures the directional-geometry gap to the local information-theoretic optimum. It should not be interpreted as the absolute performance loss caused only by the target displacement or as a direct comparison of the achieved CRLB traces of the three methods.

At an offset radius of 100m, the proposed method has a median gap of 1.71% and a 90th percentile gap of 5.07%. The corresponding values are 10.38% and 20.64% for uniform augmentation and 23.98% and 47.88% for the fixed random configuration. These results show that the proposed geometry remains close to its local information-theoretic optimum over the examined target region. The larger gaps of the reference configurations indicate that their information directions are less well matched to their respective local information structures.

## 6. Conclusions

This paper developed an analytical method for augmenting an existing two-dimensional AOA localization network with the minimum number of additional sensors under an A-optimal accuracy constraint. By representing the additional sensors as weighted vectors in a double-angle plane, the bearing-design problem was reduced to a planar vector-balancing problem. The polygon condition provided the minimum residual imbalance and A-optimal cost for fixed sensor weights. For equal-weight sensors, a direct rule for determining the required number of additional sensors and explicit bearing constructions for weak-direction compensation and tight-frame completion were obtained. The analysis also covered sensors with unequal information weights. The explicit constructions are optimal under the unconstrained bearing-design model and can provide information-theoretic performance lower bounds or initialization points when additional physical constraints, such as common-radius deployment or minimum inter-sensor separation, are imposed.

The analytical results were confirmed by numerical optimization and Monte Carlo localization experiments. The proposed design improved information balance, FIM conditioning, empirical RMSE, and tail-error performance compared with the reference geometries. Additional experiments with unequal weights, random existing networks, and target-position mismatch further supported the analysis. Future work will consider sensitivity to correlated noise, systematic bias, and non-Gaussian AOA errors, together with uncertain or multiple target positions, restricted deployment regions, three-dimensional localization, and joint sensor-type and placement design. 

## Figures and Tables

**Table 1:** Configuration of the existing AOA sensors.

Sensor	Bearing (°)	Distance (m)	*x*-Coordinate (m)	*y*-Coordinate (m)
1	5	300	298.86	26.15
2	20	320	300.70	109.45
3	40	340	260.46	218.55
4	160	380	−357.08	129.97

**Table 2:** Theoretical performance of the augmentation geometries.

Method	CRLB Trace ($m^{2}$)	CRLB RMSE (m)	Condition Number	Imbalance
Proposed frame completion	52.9723	7.2782	1.0000	0.0000
Uniform augmentation	58.4034	7.6422	1.8775	0.3049
Weak-direction concentration	58.1679	7.6268	1.8525	0.2989
Fixed random configuration	61.8449	7.8642	2.2194	0.3788

**Table 3:** Theoretical and empirical localization performance.

Method	CRLB RMSE (m)	Monte Carlo RMSE (m, 95% CI)	95% Error Quantile (m, 95% CI)
Proposed method	7.2782	7.2139 [7.1331, 7.2947]	12.6095 [12.4127, 12.8064]
Uniform augmentation	7.6422	7.6490 [7.5754, 7.7226]	13.3753 [13.1923, 13.5583]
Fixed random configuration	7.8642	7.8612 [7.7586, 7.9639]	13.8831 [13.6671, 14.0991]

## Data Availability

The original contributions presented in this study are included in the article. Further inquiries can be directed to the corresponding author.
